# Resilience in the Face of Uncertainty: Navigating Supply Chain Challenges Through Proactive Risk Surveillance and Mitigation Strategies among SMEs in ASEAN countries

**DOI:** 10.12688/f1000research.153654.1

**Published:** 2024-09-10

**Authors:** Sanmugam Annamalah, Kalisri Logeswaran Aravindan, Selim Ahmed

**Affiliations:** 1 Graduate School of Business & Research and Innovation Management Centre, SEGi University Kota Damansara, Kota Damansara, Petaling Jaya, Selangor, 47810, Malaysia; 2 Faculty of Management, Multimedia University, Cyberjaya, Selangor, 63100, Malaysia; 3 Department of Business Administration, World University of Bangladesh, Dhaka, 1230, Bangladesh

**Keywords:** Supply Chain, Risk Management, Risk Surveillance, Risk Mitigation, SMEs, ASEAN, Consumer Products

## Abstract

**Background:**

Supply chain risk management is crucial for the consumer products industry due to its vulnerability to uncertainties and risks. This study investigates the relationship between supply chain risks and performance among SMEs in the ASEAN countries. Supply chain performance, defined as meeting end-customer demands, involves ensuring commodity availability, on-time deliveries, and maintaining sufficient inventory and capacity across organizational boundaries from raw materials to the final consumer.

**Methods:**

The study utilized a sample of 385 entrepreneurs from the consumer products industry in ASEAN countries. The research was analyzed using Partial Least Squares Structural Equation Modeling (PLS-SEM) to establish the correlation between supply chain performance and risks. Factors related to the ASEAN Economic Community (AEC) for economic integration and regional trade agreements were incorporated to understand the diverse economic development, infrastructure, and regulatory environments across nations.

**Results:**

The analysis revealed a strong correlation between supply chain risks and performance. Entrepreneurs in the consumer products industry should collaborate closely with governmental organizations to address the unique challenges posed by regulatory landscapes, cross-border logistics, and geopolitical risks within the ASEAN region. Adapting to cultural nuances and market variations, along with optimizing logistics and infrastructure, are crucial for successful supply chain risk management.

**Conclusions:**

Effective supply chain risk management significantly enhances the performance of the consumer products industry’s supply chain. Entrepreneurs in this sector should align their strategies with regional and national governments to proactively address issues and mitigate risks. Continuous monitoring and adaptive measures are necessary to handle emerging risks in the dynamic market landscape. The study provides valuable insights for policymakers, suggesting that supportive frameworks and policies are necessary to bolster SME capabilities in risk management. By fostering a collaborative environment between the public and private sectors, ASEAN countries can enhance overall supply chain resilience. Future research could explore sector-specific risk management practices and their impact on supply chain robustness, underscoring the imperative for SMEs to adopt proactive and integrated risk management approaches to thrive in a complex and evolving economic environment.

## Introduction

The Association of Southeast Asian Nations (ASEAN) consumer goods industry covers various products like food, apparel, electronics, and personal care items, catering to individual consumption ( iFAST, 2023; Modisa & Jaikaew, 2022). This sector, serving a diverse and sizable population, experiences robust growth driven by economic expansion and increasing purchasing power ( Zapata et al., 2023). Emerging middle-class demographics in countries like Indonesia, Thailand, and Vietnam fuel demand ( Lee & Jones, 2023), with urbanization and e-commerce further propelling growth. Despite opportunities, competition intensifies, compounded by regulatory variations, supply chain complexities, and sustainability issues ( Pelkmans, 2023). Integral to the ASEAN economy, the consumer goods sector significantly contributes to GDP growth ( Amin & Annamalah, 2013). Projected expansion relies on rising disposable incomes and urbanization ( Irwansyah et al., 2023). However, challenges persist, including supplier disputes, logistics hurdles, and market access barriers ( Prashar, 2022). Trade liberalization fosters market consolidation, raising concerns about service quality and operational efficiency ( Ascione & Virgillito, 2023). The sector’s fragility is evident in price volatility, supply fluctuations, and consumer concerns over safety and quality ( Overbosch & Blanchard, 2023). Navigating these challenges demands a deep understanding of evolving consumer preferences and regulatory frameworks ( Isanovic et al., 2023). Adapting strategies to ensure operational resilience and quality standards is crucial for sustained success in the ASEAN consumer goods market.

### Problem statement

ASEAN SMEs in the consumer goods sector face significant supply chain risks that impact their operational resilience. These challenges include limited financial resources, making them vulnerable to disruptions like natural disasters and economic downturns ( Kurniawan et al., 2024). Reliance on a few suppliers exacerbates risks such as insolvency and quality issues ( Panchal et al., 2023). Dynamic markets with fluctuating demand further complicate supply shortages and production bottlenecks ( Annamalah et al., 2018). Additionally, limited technological capabilities hinder supply chain visibility and risk mitigation ( El-Garaihy et al., 2024), while inefficient inventory management and constrained transportation options increase vulnerability ( El Jaouhari & Hamidi, 2024). Regulatory compliance and quality issues compound these risks ( Yang et al., 2024). Furthermore, the lack of in-house expertise and a comprehensive risk management strategy heighten susceptibility ( Derouiche et al., 2021). Insufficient data and analytics capabilities impede accurate demand forecasting ( Nguyen et al., 2023), and difficulties in adopting advanced technologies and navigating complex regulations persist ( Pushp & Ahmed, 2023). Scaling to accommodate growth strains existing processes and infrastructure. Addressing these challenges necessitates effective risk management tools and strategies ( Habbal et al., 2024). However, research combining quantitative and theoretical methods is lacking in ASEAN nations ( Duong et al., 2023), and validated management procedures are needed to mitigate supply chain risks effectively ( Ha & Chuah, 2023). Dynamic consumer behavior and the evolving retail landscape present both opportunities and challenges for consumer goods manufacturers ( Raghavan et al., 2024). To adapt, companies must understand local preferences and compete with modern retail formats ( Mujianto et al., 2023). Robust e-commerce strategies leveraging improved internet access are crucial ( Shanmugalingam et al., 2023). Optimizing the supply chain for efficient logistics and differentiation through innovation is also vital ( Ivanov & Keskin, 2023; Annamalah et al., 2020). Success demands adaptability, strategic planning, and deep market understanding ( Raghavan et al., 2024).

This study aims to explore the relationship between supply chain risks and supply chain performance in the consumer products sector within ASEAN nations. Specifically, it investigates whether risk monitoring and risk mitigation serve as mediators in this relationship. The concept of “supply chain performance” in this context refers to the efficiency and effectiveness of the supply chain in meeting end-customer demands, ensuring commodity availability, on-time deliveries, and maintaining sufficient inventory and capacity across the supply network, from raw materials to the final consumer. By clarifying and differentiating these terms, the study seeks to provide a comprehensive understanding of how supply chain risk management practices can enhance performance outcomes among SMEs in the ASEAN consumer product sector.

### Objective

The primary objective of this research is to adopt a comprehensive approach to supply chain risk management to mitigate the impact of supply chain risks and enhance supply chain performance among SMEs entrepreneurs in ASEAN nations that are engaged in consumer product production. In addition, this study aims to explore the interplay between supply chain risks and supply chain performance in the consumer products sector. Specifically, it seeks to investigate whether the monitoring of risks acts as a mediator in the relationship between supply chain risk management and performance, as well as whether risk mitigation similarly mediates the relationship between supply chain risk management practices and performance outcomes among ASEAN nations.

## Literature review

### Supply Chain Performance (SCP)

Supply Chain Performance (SCP) encompasses meeting end-customer expectations, ensuring product availability, timely deliveries, and maintaining adequate inventory and capacity ( Liu et al., 2023). Performance evaluations enhance collaboration and guide strategic decisions. Socioeconomic changes, including heightened consumer expectations, reshape performance standards, demanding higher quality across various aspects ( Zaman & Kusi-Sarpong, 2024). SCP is deeply influenced by operational, financial, strategic, and external risks ( Guo et al., 2024). Operational risks include disruptions in production or logistics. Financial risks entail currency fluctuations or cost escalations. Strategic risks involve market demand changes or regulatory shifts. External risks like natural disasters or global pandemics compound complexities. Supply chain managers must continually assess, monitor, and adapt strategies to mitigate disruptions ( Umar & Wilson, 2024). Establishing resilient networks with dual sourcing and inventory optimization is essential. Collaboration among partners fosters responsiveness and agility ( Guntuka et al., 2024). Successfully integrating risk management enhances resilience and gains a competitive edge ( Guntuka et al., 2024). Adaptability and preparedness define a supply chain’s ability to deliver products or services, even amidst challenges. Heightened awareness of sustainability affects consumer preferences. Effectively managing risks involves robust mitigation strategies, technology investments, supplier collaboration, and continuous monitoring ( Kwaramba et al., 2024). A resilient supply chain overcomes uncertainties, ensuring consistent performance and meeting customer expectations.

### Significance of supply risk in the context of supply chain performance

Risk assessment in supply chain management encompasses diverse perspectives, including strategy, finance, production, accounting, and marketing ( Shelar et al., 2023). Dong’s definition characterizes risk as the fluctuation in potential supply chain outcomes, highlighting disruptions between components ( Rafi-Ul-Shan et al., 2024). Both local logistics managers and global supply chain professionals may hold distinct views on prevalent risks, which stem from unforeseen micro and macro events, leading to operational, tactical, or strategic failures ( Rafi-Ul-Shan et al., 2024). Supply chain risks manifest as internal or external, with examples like natural disasters representing external risks and concerns about supplier quality exemplifying internal risks ( Hajmohammad et al., 2024). Supplier failure is notably significant, underscoring the importance of timely order delivery, with studies showing the effectiveness of employing multiple suppliers in minimizing operational and interruption issues ( Hajmohammad et al., 2024). Porter’s value chain model emphasizes the integration of operations, logistics, and the supply chain’s success, highlighting the impact of supply risk on outbound logistics and overall efficiency ( Bustinza et al., 2024). For productivity and enterprise selection, reliable and affordable communications, energy, and information technology are vital ( Bustinza et al., 2024). Effective management of supply risk is paramount for achieving and sustaining a high level of supply chain performance, often measured by metrics like efficiency, effectiveness, and responsiveness ( Ngo et al., 2023). Any disruption within the supply chain can significantly impact these key indicators, leading to increased costs, inefficiencies, stockouts, production delays, customer dissatisfaction, and potential harm to a company’s reputation and loyalty ( Wang et al., 2024). Resilient supply chains can swiftly adapt and recover from disruptions, which can manifest in various forms, from supplier financial instability to demand fluctuations ( Negri et al., 2024). Mitigating supply risks involves strategies such as diversifying suppliers, maintaining safety stock, utilizing advanced technologies for enhanced visibility, and conducting regular risk assessments and mitigation plans ( Negri et al., 2024). In today’s intricate global supply chains, proactive management of supply risks, informed by academic research, is imperative for sustaining performance amidst uncertainties and disruptions.

H1a: There exists a significant relationship between supply risk and supply chain performance.

### Significance of supply risk in the context of risk monitoring

Internal risks, within a supplier’s control, are distinct from external risks, which lie outside their sphere, such as political instability in operating regions ( Yu et al., 2023). Both internal and external risks must be addressed in comprehensive risk management strategies ( Haraguchi et al., 2023). While some risks, like natural disasters, may be unavoidable, firms can develop strategies to mitigate their impact ( Haraguchi et al., 2023). In the realm of supply chain management, there exists a symbiotic relationship between supply risk and risk monitoring. Supply risk encompasses potential disruptions, uncertainties, and vulnerabilities within the supply chain, while risk monitoring involves the systematic tracking and assessment of these risks. The nexus between these concepts lies in the understanding that effective risk monitoring requires a comprehensive grasp of supply risks ( Berger et al., 2023). It is essential to emphasize that managing supply risk extends beyond crisis response to proactive identification and mitigation. Risk monitoring plays a pivotal role in this regard by facilitating early detection, enabling organizations to take preventive measures. For instance, if organizations identify potential disruptions, such as a supplier’s financial instability, through monitoring, they can take proactive steps like diversifying their supplier base or securing alternative sources ( Jessin et al., 2023). Supply risk is dynamic, with new risks emerging and existing ones evolving over time. Risk monitoring, as an ongoing practice, enables companies to stay attuned to shifting conditions, regulatory changes, and market dynamics affecting their supply chain. This continuous awareness empowers organizations to adapt and enhance their risk management strategies effectively. Effective risk monitoring relies on data and information, including supply chain data, market insights, and real-time intelligence ( Casagli et al., 2023). These sources feed into risk monitoring systems, facilitating informed decision-making and responsive strategy development. Additionally, risk monitoring serves as an evaluative tool for assessing the efficacy of risk mitigation strategies and making necessary adjustments. Efficient supply risk management through risk monitoring can lead to significant cost savings in the long term. By addressing risks proactively, companies can avoid the costly emergency measures associated with crisis management, ensuring smoother and more cost-effective operations ( Pham et al., 2023). In essence, supply risk is central to risk monitoring in supply chain management. Effective risk monitoring is crucial for identifying, assessing, and mitigating supply risks, thereby enhancing the resilience and efficiency of supply chain operations. These concepts work together to safeguard the continuity and stability of supply chain operations.

H1b: Supply risk has a significant relationship with risk monitoring.

### Significance of supply risk in the context of risk mitigation

In the consumer products industries, supply risk pertains to the possibility of disruptions, uncertainties, or vulnerabilities within the supply chain that could negatively affect the production, distribution, or availability of consumer goods ( Kristanto & Kurniawati, 2023). Many companies in consumer product sector heavily rely on global supply chains for sourcing raw materials, components, and finished products, exposing them to risks associated with international trade such as geopolitical tensions, currency fluctuations, and trade restrictions ( Kristanto & Kurniawati, 2023). Effective strategies to mitigate these risks are crucial to navigate uncertainties and maintain a stable supply of goods. Disruptions like transportation network issues, infrastructure damage, or production facility impacts can occur due to various events, emphasizing the importance of resilience and continuity planning ( Birge et al., 2023). Supply chain management in consumer products industries often involves intricate networks of suppliers, subcontractors, and manufacturers spanning multiple countries, necessitating thorough monitoring and understanding of these relationships to identify and address potential risks effectively ( Berger, et al., 2023). This may include actions like diversifying supplier sources, establishing contingency plans, and strengthening partnerships. Compliance management is also essential due to varying regulatory requirements across ASEAN nations and international markets, as failure to comply can lead to production and distribution disruptions, product recalls, and damage to reputation ( Kaewnuratchadasorn, 2023). Furthermore, market unpredictability, driven by fluctuating demand, evolving consumer behavior, and emerging trends, poses additional challenges, underscoring the need for strategies that incorporate market intelligence, demand forecasting, and agile supply chain practices. The COVID-19 pandemic has highlighted the vulnerabilities of today’s interconnected supply chains, among ASEAN nations, emphasizing the risks associated with just-in-time inventory management and heavy reliance on a limited number of suppliers or single-source suppliers ( Todo et al., 2023). Additionally, the digital age introduces cybersecurity risks, with cyberattacks on suppliers potentially disrupting operations and compromising sensitive data, further emphasizing the importance of proactive risk mitigation measures to safeguard supply chains and ensure operational resilience.

H1c: Supply risk has a significant relationship with risk mitigation.

### Significance of process risk in the context of supply chain performance

Process risk is a critical aspect of supply chain management, with significant implications for operational efficiency and customer satisfaction ( Prime et al., 2020). It encompasses internal disruptions within a company’s processes, impacting the flow of products and services and necessitating effective management to maintain a resilient supply chain ( Shekarian & Mellat Parast, 2021). These internal disruptions can stem from various factors such as equipment breakdowns, labor strikes, and quality issues, all of which have the potential to halt or slow down production processes and ultimately affect overall supply chain efficiency ( Shekarian & Mellat Parast, 2021). The unpredictability inherent in manufacturing systems, including variability in machine downtime and flow, introduces uncertainty into the production process, making it challenging to maintain a consistent and reliable workflow ( Park et al., 2024). Such variability can impact key performance indicators such as throughput time, process yield, and product quality, introducing inconsistencies in the manufacturing process and leading to inefficiencies ( Park et al., 2024). Process risk directly affects the throughput time of production processes, potentially resulting in delays in delivering products to customers ( Daultani et al., 2024). Moreover, quality issues arising from process risks can undermine product quality, leading to rejections, rework, and ultimately, customer dissatisfaction ( Daultani et al., 2024). Given that efficient supply chain performance relies on smooth and reliable internal processes, effectively managing process risks is crucial for maintaining operational excellence and meeting customer demands ( Daultani et al., 2024). Companies must implement robust risk management strategies to identify, assess, and mitigate process risks. This may involve investing in preventive maintenance, quality control measures, and contingency plans to address potential disruptions in the production process ( Munir et al., 2024). Despite its significance, process risk in supply chain management is sometimes overlooked compared to other challenges such as demand and supply fluctuations. Therefore, further research is needed to better understand and address process risks to enhance overall supply chain resilience and performance ( Liu et al., 2024).

H2a: Process Risk has a significant relationship with Supply Chain Performance

### Significance of process risk in the context of risk monitoring

The significance of process risk within the domain of risk monitoring is an indispensable facet of contemporary business operations as process risk denotes the potential disruptions, errors, or deviations that may manifest within an organization’s operational workflows ( Tsang et al., 2018). Effective risk monitoring serves as the linchpin for identifying, evaluating, and mitigating these risks, thereby ensuring the seamless functionality of business processes and the attainment of organizational objectives ( Pham et al., 2023). The process of risk monitoring commences with the identification of potential risks, stemming from both internal factors such as process intricacies, resource constraints, and inadequate training, as well as external influences like regulatory shifts and market fluctuations. This identification process is fundamental, as identified risks must undergo comprehensive assessment and quantification, involving ongoing evaluation to gauge their likelihood and impact on operational processes. Continuous vigilance, employing real-time data, performance metrics, and key performance indicators, forms the bedrock of effective risk monitoring ( Aljohani, 2023). However, risk monitoring transcends mere assessment as it encompasses the implementation of strategic mitigation measures such as process redesign, technological enhancements, and employee training. Furthermore, it extends its purview to encompass compliance with stringent regulatory requirements, curtailing the potential legal and financial repercussions. The role of process risk in the domain of risk monitoring is pivotal, ensuring the dependability and efficacy of organizational processes and through the continual evaluation, quantification, and mitigation of process risks, businesses fortify their operational resilience, enabling them to adeptly navigate evolving challenges ( Pham et al., 2023). A process encompasses crucial management tasks within a company, relying on locally owned resources and operational infrastructure and therefore process risks, internal to the corporation, stem from disruptions in product flow through various processes, such as equipment breakdowns, technical advances, labor strikes, and quality issues ( Mc Loughlin et al., 2023). Unpredictability in manufacturing systems includes machine downtime, operator availability, and flow variability, impacting throughput time, process yield, and product quality. Process risk threatens a producer’s ability to fulfill customer orders and compromises supply chain efficiency. While demand and supply challenges have received more attention, future research should focus on addressing process risk.

H2b: Process risk has a significant relationship with risk monitoring.

### Significance of process risk in the context of risk mitigation

The significance of process risk within the context of risk mitigation stands as a vital component of contemporary business operations as process risk entails the potential disruptions, errors, or deviations that can manifest within an organization’s operational workflows, making effective management paramount to identify, assess, and mitigate these risks ( Schuett, 2023). This ensures the seamless functionality of business processes and the realization of organizational goals. Identifying process risks involves considering a spectrum of sources, encompassing internal elements like process intricacies, resource constraints, and employee mishaps, alongside external factors such as regulatory shifts and supply chain perturbations. Methodologies for pinpointing operational risks, which often overlap with process risks, are important because they allow organizations to systematically identify vulnerabilities and potential points of failure within their operations, enabling proactive risk mitigation and ensuring the continuity and resilience of business processes ( Fang et al., 2024). It becomes crucial to quantify and assess these risks, which requires conducting a comprehensive evaluation of both their potential impact and likelihood, ensuring a thorough understanding of their potential consequences and probability of occurrence. Quantitative methodologies, such as the utilization of risk matrices and Monte Carlo simulations, are frequently employed to address this need, providing systematic frameworks for evaluating and quantifying operational and process risks by assessing both their severity and probability, thereby enhancing decision-making and risk management strategies ( Tunçel et al., 2023). Continuous monitoring and surveillance, underpinned by real-time data, key performance indicators (KPIs), and performance metrics, are essential to detect deviations and risks as they arise. Data analysis techniques for continuous monitoring are crucial for detecting trends, anomalies, and emerging risks in real-time, enabling proactive risk mitigation and ensuring timely response measures can be implemented to safeguard operations and mitigate potential disruptions. Extending beyond observation, risk monitoring extends to the implementation of mitigation strategies that may encompass process redesign, technological enhancements, training initiatives, or contingency planning ( Menz et al., 2024). Furthermore, stringent regulatory requirements, particularly in industries like finance, underscore the importance of process risk mitigation to ensure compliance. Overall, process risk occupies a pivotal role in the arena of risk mitigation, safeguarding the dependability and efficiency of organizational processes.

H2c: Process risk has a significant relationship with risk mitigation.

### Significance of demand risk in the context of supply chain performance

Demand risk significantly impacts supply chain performance, particularly for Small and Medium-sized Enterprises (SMEs) due to their size, resource limitations, and constrained capabilities ( Ali et al., 2023). SMEs operate with leaner inventory levels, making them vulnerable to inaccuracies in demand forecasting and leading to either overstocking or stockouts ( Gurbuz et al., 2023). This can result in increased carrying costs or missed sales opportunities, affecting SMEs’ financial health ( Patil & Prabhu, 2024). Fluctuations in demand strain SMEs’ financial stability, hindering operational expenses and investments in critical areas ( Patil & Prabhu, 2024). Moreover, SMEs heavily depend on a limited number of suppliers, and demand variability can strain supplier relationships, affecting production inputs ( Deng et al., 2022). Inconsistent product availability due to demand uncertainties can lead to dissatisfaction among customers, impacting brand image and loyalty ( Dewi & Hastuti, 2024). SMEs may lack the flexibility to adapt quickly to demand fluctuations, affecting production schedules and scaling operations ( AlZayani et al., 2024). Investing in affordable and user-friendly technology solutions can empower SMEs to better understand and respond to demand changes ( AlZayani et al., 2024). Recognizing the significance of demand-related challenges is essential for SMEs to develop resilient and agile supply chains that can navigate uncertainties and sustain growth ( Lu et al., 2024). Demand risk profoundly impacts various aspects of supply chain performance, including financial stability, supplier relationships, customer satisfaction, and adaptability. Managing this risk is vital for organizations to excel in supply chain performance, requiring robust demand forecasting and risk management strategies ( Lu et al., 2024).

H3a: Demand risk has a significant relationship with supply chain performance.

### Significance of demand risk in the context of risk monitoring

In contemporary business landscapes, demand risk is paramount within risk monitoring frameworks, especially in volatile markets and industries ( Tantsyura et al., 2016). Characterized by the unpredictability of customer demand, it profoundly influences a company’s revenue, production capabilities, and overall performance ( Thi Binh An et al., 2024). Effective demand risk management is crucial for businesses aiming to remain adaptable and financially stable ( Fu et al., 2023). This multifaceted risk encompasses economic oscillations, shifts in consumer preferences, competitive dynamics, and external variables like natural disasters or pandemics. Demand risk affects inventory management, production planning, and operational efficiency in supply chain management. Fluctuations in demand can significantly impact revenue and profitability, with overestimation leading to excessive inventory and underestimation resulting in missed sales opportunities ( Zhu et al., 2024). Companies must balance inventory, production capacities, and workforce management to respond to demand fluctuations. A resilient supply chain is crucial for adapting to demand oscillations and ensuring consistent product availability ( Swaminathan & Venkitasubramony, 2024). Demand forecasting models and advanced analytics aid in predicting demand patterns accurately. Market intelligence facilitates proactive adjustments to demand-related challenges ( Khan et al., 2020). High demand risk arises from significant volatility triggered by order changes, shorter product life cycles, or new item introductions. In consumer goods supply chains, demand risks can have long-term consequences, influenced by factors like unforeseen consumer demand and changes in food safety standards ( Prashar & Vijaya, 2024). Addressing demand-related risks is crucial for supply chain performance, particularly in the consumer products sector. Contemporary academic references offer insights into managing demand risk and its broader implications within industries ( Hasan et al., 2024). This study underscores the importance of understanding and mitigating demand-related risks in supply chain management.

H3b: Demand risk has a significant relationship with risk monitoring.

### Significance of demand risk in the context of risk mitigation

Demand risk’s significance within the domain of risk mitigation is paramount in contemporary business operations, especially in the ever evolving and unpredictable market landscapes of today as this risk category revolves around the intricacies of customer demand’s variability and unpredictability for products or services, exerting profound effects on revenue, production, inventory management, and overall business performance ( Khan et al., 2024). Effective management of demand risk serves as a linchpin for businesses, enabling them to navigate adaptably, allocate resources judiciously, and secure financial stability. Demand risk encompasses multifaceted origins, from economic fluctuations and shifting consumer preferences to competitive dynamics and unexpected disruptions ( Ngo et al., 2024). The financial implications of demand risk are significant, ranging from the consequences of overestimating demand, such as excessive inventory and holding costs, to the missed opportunities stemming from underestimation ( Annamalah et al., 2018). Balancing inventory levels with demand variability stands as a foundational element within demand risk management, highlighting the intrinsic relationship between demand risk and supply chain resilience ( Rohaninejad et al., 2023). This correlation underscores the significance of demand risk, as a resilient supply chain not only bolsters adaptability to demand fluctuations but also ensures the continual availability of products. Achieving this equilibrium between inventory management and demand unpredictability is essential for fostering supply chain resilience and effectively navigating market uncertainties. Employing demand forecasting models and advanced analytics is pivotal for mitigating demand risk, as accurate demand forecasts empower proactive decision-making, efficient resource allocation, and risk mitigation ( Swaminathan & Venkitasubramony, 2024). Furthermore, staying attuned to market dynamics, consumer behavior shifts, and competitive landscapes is imperative to address demand risk effectively.

H3c: Demand risk has a significant relationship with risk mitigation.

### Significance of risk monitoring in the context of supply chain performances

In contemporary business operations, risk monitoring is crucial for supply chain performance, especially in today’s globally interconnected and complex supply chain networks ( Yang et al., 2023). It involves ongoing assessment and mitigation of various risks that can disrupt supply chain operations, impacting cost-effectiveness and customer satisfaction. Effective risk monitoring allows organizations to proactively detect potential disruptions, such as natural disasters, geopolitical tensions, supplier issues, or cyber threats ( Ganesh & Kalpana, 2022), enabling the implementation of strategic measures to minimize their impact. Supply chain disruptions, such as operational downtime or missed deliveries, can adversely affect customer satisfaction. Risk monitoring plays a vital role in ensuring operational continuity by promptly addressing issues, optimizing resource allocation, and enhancing cost-efficiency ( Mohezar et al., 2023). By identifying and mitigating risks, organizations can avoid unnecessary expenses associated with disruptions, such as rushed orders, excess inventory, or production delays. Moreover, continuous risk monitoring fosters stronger relationships with suppliers through transparent communication and collaborative risk mitigation strategies, thus building a more resilient supply chain ( Hajmohammad et al., 2024). The regulatory landscape significantly influences supply chain operations, and risk monitoring helps organizations navigate regulatory changes, reducing the risk of legal and financial consequences ( Bangar et al., 2024). Effective risk monitoring ultimately translates into customer satisfaction, as an agile and efficient supply chain consistently delivers products and services on time, exceeding customer expectations.

By identifying, assessing, and mitigating various risk factors, risk monitoring optimizes supply chain performance ( Wiryasaputra et al., 2023). Continuously evaluating potential threats allows organizations to proactively adjust strategies, enhance agility, and foster collaboration across the supply chain ecosystem, ensuring operational efficiency, and effectively meeting customer demands.

H4: Risk monitoring significantly mediates the relationship between the factors and supply chain performance.

### Significance of risk mitigation in the context of supply chain performances

In contemporary business operations, risk mitigation is paramount for supply chain performance, especially in an era marked by global interconnectivity and increasing complexities ( Dohale et al., 2023). It involves formulating and implementing strategies to identify, assess, and alleviate risks, ensuring seamless flow of goods, cost-effectiveness, and customer satisfaction ( Hashim et al., 2024). Effective risk mitigation starts with systematically identifying and evaluating potential risks inherent in the supply chain, including disruptions and supplier issues ( Wiryasaputra et al., 2023). This foundational step is crucial for developing mitigation strategies. Risk mitigation extends to ensuring operational continuity by minimizing disruptions’ repercussions, requiring contingency plans, alternative suppliers, and swift risk response strategies ( Dohmen et al., 2023). It significantly contributes to cost efficiency by circumventing expenses from disruptions like urgent orders and excess inventory holdings ( Groenewald et al., 2024). Mitigating risks enhances supply chain resilience, enabling swift adaptation and recovery from disruptions ( Alsmairat, 2023). It fosters robust supplier relationships through transparent communication and collaborative risk mitigation strategies. Ultimately, effective risk mitigation reverberates in customer satisfaction, as a resilient supply chain ensures timely delivery of products and services, fostering loyalty ( Alsmairat, 2023). This interplay between risk mitigation, supply chain resilience, and customer satisfaction is pivotal for optimized supply chain performance. In implementing risk mitigation measures, precise knowledge gained through comprehensive risk assessment is crucial ( Hochrainer-Stigler et al., 2024). Success relies on accurate risk assessment, with all mitigation efforts based on data from this phase. In consumer product supply chains, where entrepreneurs face high risks and low awareness, sharing risk information is crucial for reducing risks and promoting sustainable performance ( Muttitt et al., 2023). This research emphasizes the importance of knowledge sharing in enhancing the effectiveness of risk mitigation tactics for consumer product entrepreneurs, crucial for sustainable supply chain performance.

H5: Risk mitigation significantly mediates the relationship between the factors and supply chain performance.

### Underpinning theory

**Figure 1.**
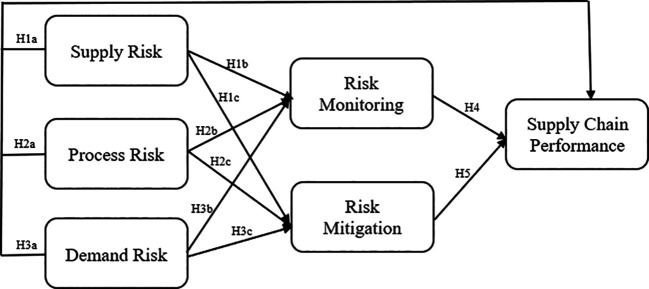
Research Framework of the Impact of Risk Variables on Supply Chain Performance Mediated by Risk Monitoring and Mitigation. Modified from various studies: Aqlan & Lam (2015); Ho et al. (2015); Kern et al. (2012).

The purpose of risk identification is fundamental to supply chain management, aiming to uncover all relevant risks and anticipate future uncertainties for proactive management. Without identifying risks, no risk management measures can be implemented. Early assessment during risk identification is crucial to determine the relevance of a risk and whether further assessment or mitigation is necessary ( Cevikbas et al., 2024). A comprehensive approach to risk identification is essential to identify all potential threats and vulnerabilities in the supply chain. Successful Supply Chain Risk Management (SCRM) requires a thorough yet swift and cost-effective evaluation of Supply Chain Risks (SCRs), which can be achieved through risk assessment utilizing available data or expert judgment and scenarios ( Chen et al., 2024). Integrating objective data with subjective perceptions enhances the robustness of risk identification, improving the accuracy of risk prediction and assessment. Prioritizing risks is crucial for organizations to focus on the most significant ones. Risks with substantial impact or those that can be promptly mitigated are assigned high priority ( Gupta et al., 2023). Risk prioritization aids in determining which risks to target with actions, enabling efficient allocation of limited resources for risk treatment ( Annamalah et al., 2019). Understanding the interconnections among risks and their cascading effects is vital for prioritizing risks, devising treatment plans, and executing effective risk management measures ( Schulman, 2023). Identifying the most crucial risk capable of triggering multiple risks is essential for effective risk management. Continuous monitoring is necessary to ensure that acknowledged risk consequences do not intensify. If consequences surpass a specified threshold, organizations must contemplate strategies to evade, transfer, share, or mitigate the risk ( Habbal et al., 2024). Mitigation aims to decrease risk to an acceptable level, addressing both the likelihood and consequences of a risk event. The choice of a mitigation strategy is influenced by the risk type, organization’s budget, and careful evaluation of acceptance, avoidance, sharing, and transfer options ( Gurbuz et al., 2023). Given the interconnected nature of risks, mitigating one type may exacerbate or alleviate another. Therefore, mitigation strategies should be applied with minimal contradictions and careful consideration of risks with negative dependencies. Investing in risk avoidance becomes essential for high probability, high impact risks, while risk acceptance may be considered for low probability, low impact risks ( Aldrighetti et al., 2023). Risk is dynamic and requires ongoing monitoring to assess the evolution of risk sources and determine whether adjustments to treatment strategies are necessary. Effective Supply Chain Risk Management (SCRM) contributes to improved supply chain performance by enhancing resilience, reducing disruptions, and ultimately improving overall performance ( Emrouznejad et al., 2023). Continuous risk monitoring, proactive risk mitigation, and effective response planning are crucial elements in achieving this objective. This model offers valuable insights into the interactions between different risk factors and their impact on supply chain resilience. Managing supply chain risks involves identifying, modeling, and mitigating risks through a framework that emphasizes continuous monitoring and real-time analytics. Adapting this model requires customizing risk identification, modifying modeling techniques, and implementing industry-specific mitigation strategies ( Aqlan & Lam, 2015; Ho et al., 2015; Kern et al., 2012). The model described herein is entirely original and has not been adapted from any previously published models. Our approach draws upon insights from several key studies, including ( Aqlan & Lam, 2015; Ho et al., 2015; Kern et al., 2012), which provided valuable foundational knowledge and data that informed the development of our model. However, the conceptual framework, structure, and implementation of the model are uniquely developed by the authors for this research. This framework aims to extend the understanding of how risk management practices influence supply chain performance as shown in Figure 1.

## Methods

This study adopts a deductive research approach within a positivist paradigm, employing quantitative methods. Deductive research starts with a general premise and derives specific conclusions through empirical observation and verification ( de Mast et al., 2023). The positivist nature of this research is reflected in its application of quantitative techniques, including surveys and structured questionnaires. Hypotheses are formulated to quantify the relationships between variables of interest, and these hypotheses are rigorously tested through statistical analysis ( Strunk, 2023). The focus is on individuals in authoritative positions within micro, small, and medium-sized enterprises (MSMEs), such as owners, executives, and managers. Primary data is collected via surveys directed at SME owners and managers across different companies. The data underpinning this study are publicly accessible in the Figshare repository ( Annamalah, 2024). The questionnaires utilize a Likert scale format. Confirmatory factor analysis is used to assess and measure the identified factors, with Partial Least Squares Structural Equation Modeling (PLS-SEM) applied to evaluate internal consistency ( Hair et al., 2023). A probability sampling method, specifically simple random sampling, was used for SME selection. Efforts were made to ensure the accurate identification of eligible respondents within the consumer product sector, including managers, owners, and senior executives. As of 2020, there are approximately 71 million SMEs in Southeast Asia ( ASEAN, 2022), representing 97% of all businesses in the region and employing 67% of the workforce ( ASEAN, 2022). SMEs are critical to economic development, job creation, innovation, and poverty reduction in Southeast Asian countries. Indonesia, with its large population and economy, has a significant number of SMEs, while Thailand and Vietnam also serve as major hubs for these enterprises. Table 1 provides details on the number of SMEs across ASEAN member states.

**Table 1.** Number of SMEs in Southeast Asia.

Countries	No of SMEs
Indonesia	64200000
Thailand	3200000
Malaysia	1173601
Philippines	1105143
Vietnam	1109684
Cambodia	512870
Singapore	299800
Laos	126717
Myanmar (Burma)	126237
Brunei	5990
Total	71860042

Source: ASEAN (2022).

### Ethical statement

We, the undersigned co-authors, affirm that our manuscript titled “Resilience in the Face of Uncertainty: Navigating Supply Chain Challenges Through Proactive Risk Surveillance and Mitigation Strategies among SMEs in ASEAN Countries” contains original material that has not been published, either in full or in part, in any other journal or publication. This manuscript is not under consideration for publication elsewhere. Each author has made significant and active contributions to the research and preparation of this manuscript and accepts joint and individual responsibility for its content. This study was conducted in accordance with the ethical principles outlined in the Declaration of Helsinki. Ethical approval for the research was obtained from the Institutional Review Board (IRB) of SEGi University with the approval number IRB-2023-14532, granted on May 5, 2023. Participants were provided with comprehensive information about the study, including its purpose, procedures, potential risks, and benefits. Written informed consent was obtained from all participants prior to their inclusion in the study. They were assured that their participation was voluntary and that they could withdraw at any time without any consequences. In cases where verbal consent was obtained due to [specific reasons, e.g., participants’ literacy levels, cultural considerations], this approach was approved by the IRB, ensuring that all participants received the necessary information and gave their informed consent voluntarily. The IRB reference number for this approval is IRB-2024-12346.

This research covers a range of countries including Singapore, Indonesia, Thailand, Malaysia, Philippines, Vietnam, Laos, Myanmar (Burma), Brunei, and Cambodia, as indicated in Table 2. Moreover, the classification of survey participants is carefully determined through comprehensive analysis of statistical data, taking into account factors such as each country’s business attractiveness, historical context, and specific business preferences or priorities ( ADB, 2022). Subsequently, the response samples from each country were further segmented for in-depth analysis.

**Table 2.** Country respondents.

Countries	No of respondents
Indonesia	94
Thailand	77
Malaysia	63
Philippines	51
Vietnam	25
Singapore	22
Laos	18
Myanmar (Burma)	13
Brunei	12
Cambodia	10
Total	385

The survey sample comprised 385 organizations spanning diverse industries across the surveyed region. These organizations were selected based on statistics provided by ADB (2022) to ensure representation from various business sectors. The objective of the survey is to gather information and insights about different industries, and the selection process aimed to include a representative sample of organizations or businesses from these sectors. The organizations were categorized into specific consumer products sectors according to their primary focus and offerings, which included Technology and Electronics, Fashion and Apparel, Beauty and Personal Care, Health and Wellness, Food and Beverage, among others (refer to Table 3).

**Table 3.** Sample categories of Companies.

Company Type	Organisations samples
Technology and Electronics	33
Fashion and Apparel	32
Beauty and Personal Care	30
Health and Wellness	29
Food and Beverage	27
Home and Kitchen Appliances	26
Toys and Games	25
Furniture and Home Decor	22
Pet Products	21
Sports and Outdoor Equipment	19
Baby and Childcare Products	19
Automotive Accessories	18
Travel and Luggage	18
Consumer Electronics Accessories	16
Jewellery and Accessories	16
DIY and Home Improvement	12
Stationery and Office Supplies	11
Crafts and Hobbies	11
Total	385

After developing the survey instrument, a preliminary evaluation was undertaken to assess its suitability and relevance. This pre-test was conducted to confirm that the questionnaire was clear and acceptable to its target audience before its implementation in the actual study, a crucial aspect of an invention survey ( Brzoska et al., 2022). To accomplish this, the questionnaire underwent systematic testing involving participation from managers, owners, and executives holding decision-making positions in SMEs, along with academic researchers. A total of 10 respondents, all of whom were managers, owners, or executives in SMEs within the business industry, were engaged in pre-test to evaluate the questionnaire’s content, readability, and completion time. Subsequently, a pilot study was conducted, involving 40 respondents, which represented approximately 10% of the anticipated sample size for the main study ( Morin, 2013). These respondents included managers, proprietors, and executives actively engaged in the operations of the various businesses. It is noteworthy that, due to issues related to non-responses to specific questions, only 385 out of the initially targeted 500 organizations yielded usable data. The response of the sample size 77 percent (see Table 4).

**Table 4.** Response from respondents.

Response	Instruments
Questionnaires distributed	500
Questionnaires returned	412
Questionnaires excluded	27
Questionnaires useable	385
Response rate	77%

Source: Estimate based on survey data.

### Reliability and validity

To ensure the accuracy and reliability of the measurement tools, we performed a reliability assessment using Cronbach’s Alpha. This assessment is vital for enhancing measurement consistency by identifying and addressing potential errors. For evaluating questionnaire validity, we employed a mix of adopted and adapted items, incorporating feedback from academic experts to ensure content validity. Positive feedback from the pilot study and the strong reliability of most scales, as shown in Table 4, led to only minor modifications to the questionnaire. Table 5 indicates that all study variables achieved Cronbach’s alpha values above the 0.70 threshold, demonstrating the reliability of the measurement instruments used. Additionally, the Smart-PLS technique, utilizing PLS-SEM for data analysis, was applied to verify the reliability of the instrument for future use with actual respondents.

**Table 5.** Reliability assessment of variables.

No	Variable	No. of Items	Cronbach’s α
1	Supply Risk	5	0.812
2	Process Risk	5	0.768
3	Demand Risk	5	0.839
4	Risk Monitoring	4	0.538
5	Risk Mitigation	4	0.786
7	Supply Chain Performance	5	0.822
8	All Items (Total)	28	0.831

Source: Hair et al. (2012).

### Population

MSMEs are vital contributors to the economic progress and advancement of ASEAN Member States. With approximately 71 million SMEs in the region, they represent a significant portion, ranging between 97.2% and 99.9% of total establishments within ASEAN Member States. These statistics underscore the pivotal role SMEs play in fostering economic and social development, as they actively engage in value-added activities, drive innovation, and promote inclusive growth by generating employment opportunities and maintaining widespread presence across various sectors ( Annamalah et al., 2016). Therefore, SMEs serve as the cornerstone of ASEAN’s economy, playing an essential role in fostering long-term and sustainable economic growth while also aiding in bridging the development divide.

### Sampling design

In this study, probability sampling, specifically the simple random sampling technique, was utilized to select participants. This method was chosen due to its practicality in obtaining a list of entrepreneurs from SMEs in the consumer goods industry.

## Analysis

### Common Method Variance (CMV)

Common method variance (CMV) is crucially assessed in self-reported questionnaires, especially when one person provides both predictor and criterion variables. The Harman single factor test is commonly used for this purpose, where each construct undergoes major component factor analysis. If one factor dominates covariance or one general component emerges, CMV exists. This method is essential due to CMV’s significant risk and infrequent replication studies. In this study, the first component accounted for 26% of variance, with no common factor identified, indicating no significant CMV. ( Podsakoff et al., 2003). Eigenvalues and Extraction Sums of Squared Loadings provide valuable insights into the effectiveness of the factor extraction process in capturing and explaining the variance in the dataset. The one-factor analysis according to Harman’s method was conducted. The un-rotated factor analysis revealed that the initial factor accounted for only 26.71% of the variance. Consequently, it was concluded that the common method bias did not pose a significant threat in the study, as this percentage falls below the 50% threshold of variance. Table 6 presents the findings of the Harman one-factor analysis for this study.

**Table 6.** Total variance explained.

Component	Initial Eigenvalues			Extraction sums of squared loadings		
	Total	% of Variance	Cumulative %	Total	% of Variance	Cumulative %
1	12.21	26.712	26.712	12.21	26.712	26.712
2	3.589	11.965	5.664			
3	2.558	8.526	6.193			
4	1.999	6.665	6.855			
5	1.309	4.365	7.219			

### Descriptive analysis

Table 7 displays the data’s descriptive statistics as well as the variables’ reasons. The items’ standard deviations varied from 0.63 to 0.92, while the mean ranged from 3.84 to 4.25. The mean and standard deviation figures were within an acceptable range, indicating that most participants agreed with the claims.

**Table 7.** Descriptive statistics (n=385).

Constructs	Mean	Std. Deviation
Supply Risk	4.25	0.63
Process Risk	4.08	0.61
Demand Risk	3.84	0.92
Risk Monitoring	4.09	0.63
Risk Mitigation	4.05	0.68
Supply Chain Performance	4.21	0.65

### Data normality via skewness and kurtosis

The highest values were -0.19 for skewness and 0.41 for kurtosis, respectively. According to Bajpai (2011), when employing SEM, acceptable skewness values range from -3 to +3, whereas acceptable kurtosis values range from 10 to +10. The findings demonstrate that the distribution data in this instance are essentially homogeneous Table 8.

**Table 8.** Normality Via Skewness and Kurtosis.

Constructs	Skewness	Std. Error	Kurtosis	Std. Error
Supply Risk	-0.788	0.172	0.410	0.342
Process Risk	-0.311	0.172	-0.489	0.342
Demand Risk	-0.735	0.172	0.173	0.342
Risk Monitoring	-0.421	0.172	-0.037	0.342
Risk Mitigation	-0.195	0.172	-0.552	0.342
Supply Chain Performance	-0.646	0.172	0.287	0.342

Source: Bajpai (2011).

### Measurement model analysis

In assessing the internal consistency and reliability of a measure, it’s essential for Factor Loading, Cronbach’s alpha and composite reliability (CR) to equal or exceed 0.70, and average variance extracted (AVE) to equal or surpass 0.50 ( Hair et al., 2017). The measurement model’s findings in this study reveal that all variables meet these criteria, with Factor Loading, Cronbach’s alpha and CR values surpassing 0.7, and AVE values exceeding 0.5 (see Table 9). These results indicate satisfactory reliability and internal consistency for the variables under consideration.

**Table 9.** Measurement item model assessment.

Construct	Item No	Factor Loading	Cronbach Alpha	CR	AVE
Demand Risk	DR1	0.785	0.916	0.937	0.750
	DR2	0.857			
	DR3	0.899			
	DR4	0.898			
	DR5	0.866			
Process Risk	PR1	0.846	0.950	0.961	0.833
	PR2	0.951			
	PR3	0.925			
	PR4	0.907			
	PR5	0.932			
Risk mitigation	RM1	0.833	0.831	0.887	0.664
	RM2	0.833			
	RM3	0.829			
	RM4	0.763			
Risk monitoring	RMI2	0.845	0.863	0.908	0.712
	RMI3	0.876			
	RMI4	0.887			
	RMI5	0.761			
Supply Risk	SR1	0.804	0.839	0.885	0.607
	SR2	0.800			
	SR3	0.738			
	SR4	0.788			
	SR5	0.767			
Supply Chain Performance	SCP1	0.825	0.876	0.910	0.669
	SCP2	0.859			
	SCP3	0.791			
	SCP4	0.810			
	SCP5	0.802			

**Note:** Items RM1 & RMI5 were deleted due to low loadings. CA=Cronbach’s α; CR=Composite Reliability, and AVE= Average of variance extracted; Supply Risk (SR), Process Risk (PR), Demand Risk (DR), Risk Monitoring (RM), Risk Mitigation (RMI), and Supply Chain Performance (SCP).

### Discriminant validity (Fornell–Larcker criterion)

The Fornell-Lacker criterion method for assessing discriminant validity compares the square root of the Average Variance Extracted (AVE) for each latent variable with the correlations between latent variables ( Hair et al., 2014). This method ensures that each latent variable primarily explains the variance within its own indicators rather than the variance of other latent variables. Consequently, in Table 10, the bolded diagonal elements (representing the square root of AVE) are consistently larger than the corresponding off-diagonal elements within their respective rows and columns, affirming discriminant validity.

**Table 10.** Fornell–Larcker criterion.

	DR	PR	RM	RMI	SCP	SR
DR	**0.866**					
PR	0.308	**0.913**				
RM	0.425	0.525	**0.815**			
RMI	0.371	0.372	0.753	**0.844**		
SCP	0.442	0.484	0.583	0.654	**0.818**	
SR	0.360	0.581	0.481	0.512	0.682	**0.779**

### Heterotrait-Monotrait (HTMT)

In addressing concerns related to discriminant validity within variance-based structural equation modeling, the heterotrait-monotrait ratio (HTMT) was introduced by Henseler et al. (2016) and supported by Voorhees et al. (2016). HTMT assesses the ratio of correlations within a trait to those between traits. According to Henseler et al. (2016), a benchmark value below 0.90 is recommended for evaluating discriminant validity using the HTMT criterion. As emphasized by Hair et al. (2017), discriminant validity is confirmed when HTMT values fall below 0.90. Elevated HTMT values suggest potential issues with discriminant validity, underscoring the importance of maintaining values below 0.9, as proposed by Henseler et al. (2015) and Henseler et al. (2016). Table 11 displays the HTMT values for all latent variables, indicating values lower than 0.9.

**Table 11.** Heterotrait-monotrait (HTMT).

	DR	PR	RM	RMI	SCP	SR
DR						
PR	0.323					
RM	0.485	0.582				
RMI	0.418	0.407	0.893			
SCP	0.492	0.525	0.678	0.752		
SR	0.413	0.633	0.561	0.593	0.788	

Note: Supply Risk (SR), Process Risk (PR), Demand Risk (DR), Risk Monitoring (RM), Risk Mitigation (RMI), and Supply Chain Performance (SCP).

**Figure 2.**
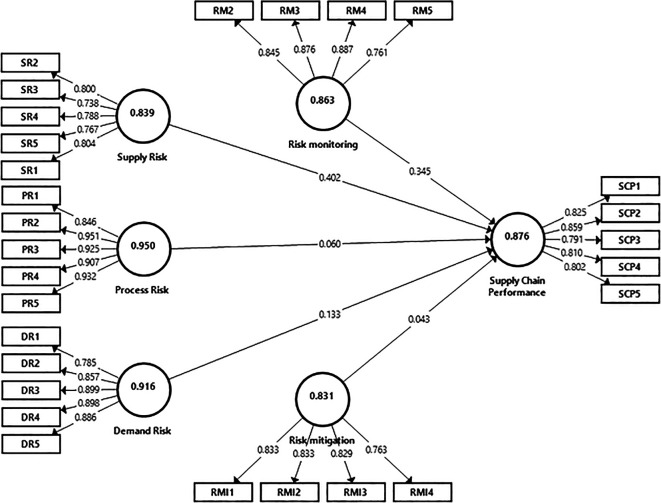
Measurement model illustration using PLS Algorithm. Source: Authors' original data.

**Structural Model** With the successful verification of the measurement model, attention can now shift towards confirming the structural model. This verification process is crucial for assessing the relationships between the constructs in the study model. model. The structural model elucidates these relationships, showcasing the interconnectedness of its components. Various measures, as outlined by Henseler et al. (2016), were employed to evaluate the degree of interconnectedness within the structural indications within the structures were established reflectively. A common approach to assessing structural models is detailed in the table provided below. The PLS (Partial Least Squares) algorithm is a statistical technique used in Structural Equation Modeling (SEM) to estimate complex cause-effect relationship models with latent variables. It focuses on maximizing the explained variance of the dependent constructs by the independent constructs. In the measurement model, PLS assesses the relationships between latent variables (unobserved constructs) and their corresponding manifest variables (observed indicators). This involves evaluating the reliability and validity of the indicators in representing the latent constructs. PLS is particularly useful in exploratory research with small sample sizes and when the primary goal is prediction rather than confirmation as stipulated in Figure 2 ( Hair et al., 2017; Wong, 2013).

A diagram depicting Structural Equation Modeling showcases the assessment of supply chain performance through independent variables such as supply, process, demand risk capital, wherein risk monitoring and risk mitigation acts as a mediator. As illustrated in Figure 2, to enhance the reliability and validity of the constructs, items with low standardized loading factors (<0.7) were excluded.

### Lateral Collinearity Assessment Test

The assessment of lateral multicollinearity requires examining the inner Variance Inflation Factor (VIF) values of the independent variables. These values should ideally be below 5 and 3.3, as recommended by Hair et al. (2016) and Tabachnick and Fidell (2023). If these thresholds are met, it suggests no significant concerns regarding multicollinearity. Furthermore, to ascertain the absence of common method bias, all VIFs resulting from a comprehensive collinearity test should be below 5, as indicated in Table 12.

**Table 12.** Lateral Collinearity Assessment Test (VIF).

	Risk mitigation	Risk monitoring	Supply Chain Performance
Demand Risk	1.169	1.169	1.273
Process Risk	1.536	1.536	1.770
Risk mitigation			2.845
Risk monitoring			2.559
Supply Chain Performance	1.597	1.597	1.824

### Assessing Goodness-of-Fit Indices

Standardized Root Mean Square Residual (SRMR) lower values indicate better fit and common thresholds for good fit range from 0.05 to 0.08. Degree of Unweighted Least Squares (d_ULS) lower values suggest better model fit. There isn’t a widely accepted threshold for d_ULS, but it should be close to zero. Degree of Geodesic Discrepancy (d_G) lower values indicate better fit ( Kenny et al., 2015). A value close to zero is desirable. NFI values range from 0 to 1, where values closer to 1 indicate better fit. Commonly, a threshold of 0.90 or higher is considered indicative of good fit. By summarizing each index and comparing the obtained values to established thresholds, it can be confidently concluded that the model exhibits a good fit to the data Table 13.

**Table 13.** Model fit (Full Summary).

	Saturated model	Estimated model
SRMR	0.028	0.033
d_ULS	0.094	0.070
d_G	0.006	0.093
Chi-Square	219.654	252.220
NFI	0.721	0.683

Source: Kenny et al. (2015).

### R square (r ^2^)

Table 14 provides evidence that the R2 values for the variables Risk Mitigation, Risk Monitoring, and Supply Chain Performance are, respectively, 0.831, 0.863, and 0.876.

**Table 14.** R ^2^ summary.

	R ^2^	Adjusted R ^2^
Risk mitigation	0.831	0.829
Risk monitoring	0.863	0.861
Supply Chain Performance	0.876	0.876

### Effect size (f ^2^)

To evaluate the magnitude of impact for each effect within the route model, researchers can employ F-square ( Table 15) following Henseler’s (2018) recommendations. The effect size, denoted as f ^2^, is calculated by comparing the increase in R-squared to the proportion of unexplained variation in the endogenous latent variable. Henseler (2018) proposes that values of 0.35, 0.15, and 0.02 signify significant, moderate, and negligible effect sizes, respectively, according to the suggested criteria for magnitude assessment.

**Table 15.** Effect size f ^2^.

	Risk mitigation	Risk monitoring	Supply Chain Performance
Demand Risk	0.387	0.352	0.436
Process Risk	0.005	0.006	0. 115
Risk mitigation			0.002
Risk monitoring			0.118
Supply Chain Performance	0.039	0.138	0.232

### Assessment of predictive relevance (Q ^2^)

The cross-validated redundancy metric (Q ^2^) serves as a crucial indicator of predictive relevance within the framework of assessment recommended by Hair et al. (2011). This metric reflects the model’s predictive capability, with values anticipated to surpass zero, signaling the model’s aptness for prediction (see Table 16 for reference). Q-square values exceeding 0.20 or 0.25 are regarded as indicative of moderate to good predictive relevance. Researchers often rely on these thresholds to evaluate the model’s effectiveness in forecasting outcomes and informing decision-making processes.

**Table 16.** Construct Cross-Validated Redundancy.

	SSO	SSE	Q ^2^ (=1-SSE/SSO)
Risk mitigation	1172.00	893.76	0.237
Risk monitoring	1172.00	926.75	0.209
Supply Chain Performance	1465.00	884.90	0.396

Note: Q ^2^ < 0

### The path coefficient

**Figure 3.**
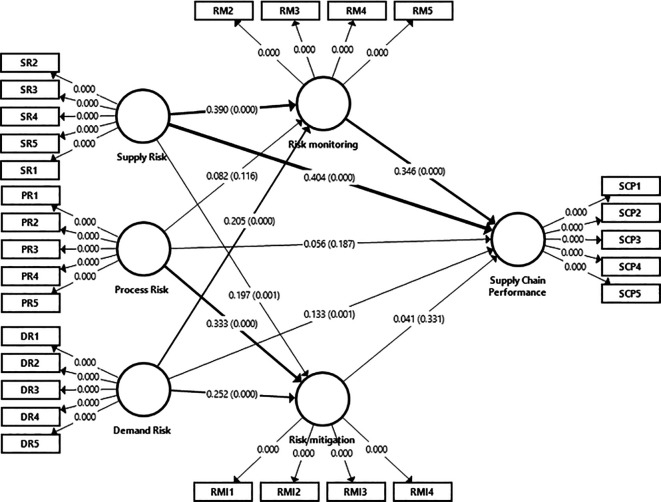
Structural model (Bootstrapping). Source: Authors' original data.

In evaluating the path coefficients linking constructs, the aim was to assess their support for the proposed hypotheses and the structural model. According to Hair et al. (2016), a minimum route coefficient value of 0.100 is deemed necessary for an impact to be considered within the model. Path coefficients were scrutinized to determine the relevance of the tested hypotheses between the constructs. Utilizing Smart-PLS bootstrapping and T-statistical analysis, the significance level of each route was established as shown in Figure 3. In the study, which involved a sample size of 385 responders and straightforward assumptions, significant values were observed at the 0.05 level. Examination of the path coefficient in Figure 3 and Table 17 unveiled relationships corresponding to hypotheses (H1a, H1b, H1c, H2c, H3a, H3b, H3c, H4) with significant values at the 0.05 level, whereas hypotheses H2a, H2b, and H5 were deemed non-significant.

**Table 17.** Path coefficient assessment.

Hypo	Relationships	*Beta*	*SD*	*t value*	*p value*	*Findings*
H1a	Supply Risk -> Supply Chain Perf	0.404	0.067	5.835	0.000	
H1b	Supply Risk -> Risk Monitoring	0.390	0.089	3.584	0.000	
H1c	Supply Risk -> Risk Mitigation	0.197	0.085	4.923	0.001	
H2a	Process Risk -> Supply Chain Perf	0.056	0.391	1.278	0.187	Not Supported
H2b	Process Risk -> Risk Monitoring	0.082	0.269	1.396	0.116	Not Supported
H2c	Process Risk -> Risk mitigation	0.333	0.057	3.981	0.000	*Supported* **
H3a	Demand Risk -> Supply Chain Perf	0.133	0.063	3.769	0.001	*Supported* **
H3b	Demand Risk -> Risk Monitoring	0.205	0.061	5.469	0.000	*Supported* **
H3c	Demand Risk -> Risk mitigation	0.252	0.051	4.923	0.000	*Supported* **
H4	Risk Monitoring -> Supply Chain Perf	0.346	0.085	4.217	0.000	Supported **
H5	Risk mitigation -> Supply Chain Perf	0.041	0.495	1.087	0.331	Not Supported

** Supported= P value <0.05.

Bootstrapping in the context of the PLS structural model is a resampling technique used to estimate the precision of the model’s parameter estimates. It involves repeatedly sampling (with replacement) from the data set and estimating the model for each sample. This process generates a distribution of estimates, which can be used to assess the stability and reliability of the path coefficients, loadings, and other statistics in the model. Bootstrapping provides confidence intervals and significance testing for the structural paths, helping researchers determine whether the relationships between constructs are statistically significant as shown in Figure 3 ( Hair et al., 2017; Henseler et al., 2009).

## Discussion

### Supply chain risks associated with consumer products

Businesses in the consumer goods sector recognize supplier, process, and demand risks as pivotal challenges shaping their supply chains ( Qiao & Zhao, 2023). Process risks arise from logistics inadequacies, inaccurate forecasting, and reliance on labor agreements. Supply and demand risks can disrupt operations due to discrepancies between supply and market demand. Weather’s impact on the economy, coupled with government regulations, exposes workplaces to environmental concerns, affecting production, sales, and transportation. Consumer goods entrepreneurs face heightened demand and financial risks compared to contract counterparts, attributed to limited resources ( Prabhu and Srivastava, 2023). Financial disruptions profoundly affect business operations, emphasizing the need for tailored financial solutions, especially for SMEs. The study reiterates the severity of supply chain risks, shedding light on their diverse impacts, underscoring the imperative for effective risk management strategies in the consumer goods sector.

### Supply chain risks and supply chain performance in consumer products

The second research objective of this study focuses on exploring the correlation between supply chain risks and supply chain performance within the consumer products sector. The findings underscore the significant impact of supply chain efficiency and risk on the ASEAN consumer products industry. The study addresses an empirical gap by establishing a clear link between supply chain risks and performance. While similar studies have made comparable assertions regarding this relationship, the research stands out for its robust analysis, particularly within the often-overlooked sector of consumer products ( Aghabegloo et al., 2024). Previous research predominantly concentrated on the manufacturing industry and primarily assessed process risks using metrics such as variance, lead time order fulfillment, and production specification ( Bokrantz & Dul, 2023; Rashid et al., 2024). However, process risk has received comparatively less attention than other forms of risk and warrants expansion from supplier-centric assessments to encompass the entire supply chain, extending from enterprises to retailers. In this study, the evaluation of supply risk was broadened to encompass foreign suppliers, yielding valuable insights into its impact on the quality, cost-efficiency, flexibility, and responsiveness of consumer products entrepreneurs. Thus, this research provides a comprehensive understanding of how supply chain risks influence overall performance within the consumer products industry.

### Risk monitoring mediates the relationship between management of supply chain risks and performance

The research findings highlight the crucial mediating role of risk monitoring in the relationship between risk management and supply chain performance. Rooted in the transaction cost concept, it’s evident that risk emerges from uncertain conditions. This study underscores that existing supply chain risks exert a substantial influence on the overall functionality of the supply chain, particularly within the highly unpredictable landscape of consumer products. Businesses operating in the consumer goods sector, are vulnerable to supply chain risks due to factors like high perishability, short shelf life, and complex engagement with various suppliers and purchasers ( Prashar & Vijaya, 2024). Effective risk management becomes imperative for enhancing supply chain performance in the face of significant supply chain risks. One of the primary objectives of this research was to examine the relationship between supply chain risks and the practices of supply chain risk management. In doing so, the study integrated perspectives from both the supply chain governance structure and the transaction cost theory ( Ülkü et al., 2024). According to the transaction cost theory, there exists a strong connection between governance systems and the levels of uncertainty within a supply chain. This research delved into assessing supply chain risks within the framework of three key risk management pillars: risk identification, risk assessment, and risk mitigation approaches. Despite the acknowledged challenges in risk detection skills among entrepreneurs in the consumer products sector, the findings of this study reaffirm the significant impact of supply chain risks on business operations ( Altay & Pal, 2023).

### Risk mitigation mediates the relationship between management of supply chain risks and performance

The research outcomes also suggest a notable mediating role of risk mitigation in bridging the link between performance and supply chain risk management. The findings highlight that among the three components comprising supply chain risk management, only risk assessment and risk mitigation act as mediators in the relationship between supply chain risk and performance. Conversely, it was observed that risk mitigation did not exert a mediating influence on supply chain performance. The investigation reveals that merely identifying risks within the supply chain doesn’t positively affect performance; instead, it’s the evaluation of risks and the implementation of mitigation measures that lead to improved performance ( Han & Um, 2024; Li et al., 2024). This sheds light on why the identification phase of risk management might not efficiently establish the link between supply chain risk and performance. Previous research on supply chain risk has often overlooked or lacked theoretical justification for incorporating risk management strategies as mediating factors ( Altay & Pal, 2023). However, the current study supports the hypothesis that effective supply chain risk management serves as an intermediary to enhance business efficiency. This notion is theoretically and empirically validated by the study, demonstrating that elements of supply chain risk management, particularly risk assessment and mitigation strategies, significantly mediate the relationship between supply chain risks and performance, specifically within the realm of consumer products entrepreneurship ( Shishodia et al., 2023).

### Theoretical implication

Supply Risk includes risks related to supplier reliability, quality issues, disruptions, etc. Theoretical contributions from SCM theory suggest that managing supply risks effectively can lead to improved operational performance, cost efficiency, and resilience in the supply chain. Process risk includes process inefficiencies, breakdowns, bottlenecks, etc. Theoretical contributions may suggest that effective process management leads to improved agility, flexibility, and responsiveness within the supply chain, contributing to overall performance ( Bokrantz & Dul, 2023). Demand risk pertains to uncertainties and variability in customer demand. Fluctuations in demand patterns, unexpected changes in customer preferences, or market dynamics can all contribute to demand risk. Theoretical contributions highlight the importance of demand forecasting, customer relationship management, and market intelligence in mitigating demand risks and improving supply chain performance ( Nurdiana et al., 2023). Risk monitoring involves the continuous surveillance and assessment of various risks within the supply chain. It includes activities such as data collection, analysis, early warning systems, etc ( Jeong & Chung, 2023). Theoretical contributions suggest that proactive risk monitoring enables timely identification of potential threats, facilitating prompt decision-making and risk mitigation actions. Risk mitigation refers to the strategies and actions taken to reduce the impact or likelihood of risks materializing within the supply chain. This can include diversification of suppliers, inventory buffers, contingency planning, etc. SCM theory emphasizes the importance of risk mitigation as a proactive approach to safeguarding supply chain performance and resilience. Theoretical contributions in SCM may emphasize that effective risk management practices positively influence supply chain performance by enhancing operational efficiency, reducing disruptions, improving customer service levels, and ultimately, contributing to competitive advantage and organizational success ( Rimin et al., 2021). SCM theory underscores the significance of addressing supply, process, and demand risks within the supply chain context and highlights the mediating role of risk monitoring and risk mitigation in enhancing supply chain performance ( Solijonov et al., 2023). By empirically testing this model, researchers can further validate the theoretical underpinnings and provide practical insights for managers to better manage risks and improve supply chain performance in dynamic and uncertain environments. Additionally, this model emphasizes the importance of a holistic and integrated approach to risk management, aligning with the overarching goals of SCM theory.

### Practical implication

By categorizing risks into supply, process, and demand categories, supply chain managers can better understand and prioritize potential threats to their operations. This allows for a more targeted approach to risk management, ensuring that resources are allocated efficiently to mitigate the most critical risks ( Halkier & James, 2022). Recognizing risk monitoring and mitigation as mediating variables underscores their importance in proactive risk management. Practically, this implies implementing robust systems for monitoring supply chain activities, gathering relevant data, and utilizing advanced analytics tools for risk detection and assessment. It also involves developing and implementing effective risk mitigation strategies tailored to the specific types of risks identified, such as diversifying suppliers, establishing contingency plans, or investing in technology to improve process resilience ( Li et al., 2024). The ultimate goal of supply chain management is to enhance performance, and the model acknowledges this by considering supply chain performance as the dependent variable. Practically, supply chain managers can use performance metrics such as on-time delivery, inventory turnover, and customer satisfaction to assess the effectiveness of their risk management efforts. By continually monitoring and evaluating performance in relation to risk management activities, managers can identify areas for improvement and implement targeted initiatives to optimize supply chain performance. The model suggests a strong coordination among supply chain partners and stakeholders, emphasizing the importance of collaboration in risk assessment and management. Practically, this involves fostering open communication channels, sharing information and insights, and establishing collaborative relationships with suppliers, customers, and other key stakeholders ( Meltzer, 2024). By working together to identify and address risks, supply chain partners can enhance their collective resilience and responsiveness to potential disruptions. Effective risk management practices contribute to building resilience and sustainability within the supply chain. By proactively identifying and mitigating risks, supply chain managers can minimize the impact of disruptions, improve operational stability, and ensure continuity of supply. This not only enhances short-term performance but also strengthens the long-term viability and competitiveness of the supply chain. Overall, the model provides a practical framework for supply chain managers to assess, monitor, and mitigate risks effectively, ultimately leading to improved supply chain performance and resilience in dynamic and uncertain environments. By aligning with supply chain management theory, it offers actionable insights and guidelines for practitioners to enhance their risk management practices and drive competitive advantage in today’s complex business landscape. Business owners in the consumer products industry may use these specifics as a reference to better understand their supplier networks. Entrepreneurs in the consumer products industry need to acquire, transform, and use knowledge in order to compete in the market. Owners of consumer products companies who have open lines of communication with their supply chain partners will be better able to identify obstacles and quickly adapt their strategy to deal with supply chain risks ( Pangestuti et al., 2023). Lowering risk will influence tactics and boost customer happiness, both of which will enhance the productivity of business owners of consumer products firms.

## Conclusion

In conclusion, the study underscores the significant impact of supply chain risks on the performance of consumer product entrepreneurs. It highlights the crucial role of supply chain risk management, particularly risk assessment and mitigation measures, in mitigating these risks and enhancing performance. Moreover, the study identifies risk monitoring and risk mitigation as mediating factors in the relationship between supply chain risks and supply chain performance. However, they do not mediate the relationships between process risk and supply chain performance, process risk and risk monitoring, as well as risk mitigation and supply chain performance. Overall, the findings emphasize the importance of continuous improvement in supply chain risk management for entrepreneurs in the consumer products industry. By focusing on and refining key elements of supply chain risk management, entrepreneurs can enhance the performance of their consumer products in the face of unpredictable supply chain dynamics. The study presents a comprehensive model for supply chain risk management tailored to the unique challenges faced by consumer product entrepreneurs, offering valuable insights for navigating and mitigating supply chain risks effectively.

### Limitations

The diverse industries and supply chain structures across ASEAN member states pose challenges, with unique economic, regulatory, and infrastructural characteristics influencing supply chain dynamics differently. Limited availability and reliability of data hinder accurate risk assessment and effective SCM strategies. Cultural and language barriers complicate data collection and analysis, while diverse consumer products and market dynamics make risk identification challenging. Informal economies introduce complexities such as counterfeit products and labor exploitation. The fragmented nature of supply chains and prevalence of small enterprises limit effective networking. Cross-border trade complexities hinder collaboration and information sharing, compromising risk mitigation efforts, especially for transnational risks like natural disasters and political instability.

### Suggestions for future research

Future research on supply chain management (SCM) in ASEAN countries should prioritize identifying and prioritizing critical risks across geopolitical, environmental, economic, and technological domains. Studies should analyze individual member states’ challenges and assess the impact of regional integration efforts like the ASEAN Economic Community (AEC) on supply chain vulnerabilities. Utilizing diverse methodologies, researchers can tailor risk assessment frameworks to ASEAN’s varied economies. Collaborative research initiatives involving academia, industry, and government can exchange best practices in risk mitigation. Exploring evolving consumer preferences, market trends, and regulatory frameworks is crucial, as is studying successful risk mitigation practices in the consumer goods industry. Research on government policies, industry standards, and international collaborations in promoting supply chain resilience is needed. Comparative analyses can identify best practices for building resilient supply chains in ASEAN. Longitudinal studies tracking supply chain risks in response to geopolitical tensions, trade disruptions, and technological advancements are also warranted. By considering internal and external risk factors, researchers can offer recommendations for enhancing ASEAN’s consumer products industry’s risk management capabilities.

## Ethical approval statement

Hereby, I, Kalisri Logeswaran Aravindan, consciously assure that for the manuscript Resilience in the Face of Uncertainty: Navigating Supply Chain Challenges Through Proactive Risk Surveillance and Mitigation Strategies among SMEs in ASEAN countries the following is fulfilled:

1) This material is the authors own original work, which has not been previously published elsewhere.
2) The paper is not currently being considered for publication elsewhere.
3) The paper reflects the author’s own research and analysis in a truthful and complete manner.
4) The paper properly credits the meaningful contributions of co-authors and co-researchers.
5) The results are appropriately placed in the context of prior and existing research.
6) All sources used are properly disclosed (correct citation). Literally copying of text is indicated by using quotation marks and giving proper reference.
7) All authors have been personally and actively involved in substantial work leading to the paper and will take public responsibility for its content.

The violation of the Ethical Statement rules may result in severe consequences.

This study has been endorsed by SEGi (RMIC) approval committee was obtained from the Institutional Review Board (IRB) of SEGi University with the approval number IRB-2023-14532, granted on May 5, 2023.

1. Sanmugam Annamalah
2. Kalisri Logeswaran Aravindan
3. Selim Ahmed

We agree with the above statements and declare that this submission follows the policies of as outlined in the Guide for Authors and in the Ethical Statement.

Date: 11/5//2023

Corresponding author’s signature: Kalisri Logeswaran Aravindan

## Ethical statement

We, the undersigned co-authors, affirm that our manuscript titled “Resilience in the Face of Uncertainty: Navigating Supply Chain Challenges Through Proactive Risk Surveillance and Mitigation Strategies among SMEs in ASEAN Countries” contains original material that has not been published, either in full or in part, in any other journal or publication. This manuscript is not under consideration for publication elsewhere. Each author has made significant and active contributions to the research and preparation of this manuscript and accepts joint and individual responsibility for its content.

This study was conducted in accordance with the ethical principles outlined in the Declaration of Helsinki. Ethical approval for the research was obtained from the Institutional Review Board (IRB) of SEGi University with the approval number IRB-2023-14532, granted on May 5, 2023.

Participants were provided with comprehensive information about the study, including its purpose, procedures, potential risks, and benefits. Written informed consent was obtained from all participants prior to their inclusion in the study. They were assured that their participation was voluntary and that they could withdraw at any time without any consequences.

The IRB reference number for this approval is IRB-2023-14532.

## Consent to participate

Informed verbal consent was obtained from all participants involved in this study. Verbal consent was deemed appropriate due to participants’ literacy levels, cultural considerations, etc. However participants received comprehensive information about the study’s purpose, procedures, potential risks, and benefits. They were assured of their right to withdraw at any time without any repercussions.

Date: 11/5/2023

**Corresponding author:** Kalisri Logeswaran Aravindan

## Participant consent form

Research Project Title: Resilience in the Face of Uncertainty: Navigating Supply Chain Challenges Through Proactive Risk Surveillance and Mitigation Strategies among SMEs in ASEAN countries

To be completed by the participant.

**Table:** 

• I have read the information sheet about this study. • I have had an opportunity to ask questions and discuss this study. • I have received satisfactory answers to all my questions. • I have received enough information about this study. • I understand that I am/the participant is free to withdraw from this study: ○ At any time (until such date as this will no longer be possible, which I have been told) ○ Without giving a reason for withdrawing • I understand that my research data may be used for a further project in anonymous form, but I am able to opt out of this if I so wish, by ticking here. • I agree to take part in this study.	
Signed (participant)	Date
Name in block letters	
Signed	Date
Name in block letters	
Signature of researcher	Date
This project is supervised by:	
Researcher’s contact details (including telephone number and e-mail address):	

## Data Availability

**Figshare:** Resilience in the Face of Uncertainty: Navigating Supply Chain Challenges Through Proactive Risk Surveillance and Mitigation Strategies among SMEs in ASEAN countries, https://doi.org/10.6084/m9.figshare.25683687.v3 ( Annamalah, 2024). **Data license:** CC BY 4.0
