# Resilience in the Face of Uncertainty: Navigating Supply Chain Challenges Through Proactive Risk Surveillance and Mitigation Strategies among SMEs in ASEAN countries

**DOI:** 10.12688/f1000research.153654.2

**Published:** 2025-06-24

**Authors:** Sanmugam Annamalah, Kalisri Logeswaran Aravindan, Selim Ahmed

**Affiliations:** 1 Graduate School of Business & Research and Innovation Management Centre, SEGi University Kota Damansara, Kota Damansara, Petaling Jaya, Selangor, 47810, Malaysia; 2 Faculty of Management, Multimedia University, Cyberjaya, Selangor, 63100, Malaysia; 3 Department of Business Administration, World University of Bangladesh, Dhaka, 1230, Bangladesh

**Keywords:** Supply Chain, Risk Management, Risk Surveillance, Risk Mitigation, SMEs, ASEAN, Consumer Products

## Abstract

Supply chain risk management is crucial for the consumer products industry, given its susceptibility to uncertainties and risks. This research investigates the relationship between supply chain risks and performance within this sector, based on a sample of 385 entrepreneurs from Small Medium Sized enterprises (SMEs) in the consumer goods industry across ASEAN countries. Supply chain performance, defined as the ability to meet end-customer demands, involves ensuring commodity availability, on-time deliveries, and maintaining sufficient inventory and capacity across organizational boundaries, from raw materials to the final consumer. Using Partial Least Squares Structural Equation Modeling (PLS-SEM), the study establishes a strong correlation between supply chain performance and risks, highlighting the necessity for effective supply chain risk management to enhance overall performance in the consumer products industry. Understanding the diverse economic development, infrastructure, and regulatory environments across ASEAN nations is crucial. Leveraging the ASEAN Economic Community (AEC) for economic integration and regional trade agreements can mitigate supply chain risks and improve performance. Entrepreneurs in the consumer products industry are encouraged to collaborate with governmental organizations, considering the challenges posed by regulatory landscapes, cross-border logistics, and geopolitical risks within ASEAN. Adapting strategies to the specific characteristics of each ASEAN country, optimizing logistics, and addressing cultural nuances are essential for successful supply chain risk management. By aligning with regional and national governments, businesses can proactively address risks, seize opportunities, and contribute to the resilience and growth of the ASEAN consumer products industry.

## Introduction

The Association of Southeast Asian Nations (ASEAN) consumer goods industry covers various products like food, apparel, electronics, and personal care items, catering to individual consumption ( Isanovic et al., 2023). This sector, serving a diverse and sizable population, experiences robust growth driven by economic expansion and increasing purchasing power ( Zapata et al., 2023). Emerging middle-class demographics in countries like Indonesia, Thailand, and Vietnam fuel demand ( Lee & Jones, 2023), with urbanization and e-commerce further propelling growth. Despite opportunities, competition intensifies, compounded by regulatory variations, supply chain complexities, and sustainability issues ( Pelkmans, 2023). Integral to the ASEAN economy, the consumer goods sector significantly contributes to GDP growth ( Amin & Annamalah, 2013). Projected expansion relies on rising disposable incomes and urbanization ( Irwansyah et al., 2023). However, challenges persist, including supplier disputes, logistics hurdles, and market access barriers ( Prashar, 2022). Trade liberalization fosters market consolidation, raising concerns about service quality and operational efficiency ( Ascione & Virgillito, 2023). The sector’s fragility is evident in price volatility, supply fluctuations, and consumer concerns over safety and quality ( Overbosch & Blanchard, 2023). Navigating these challenges demands a deep understanding of evolving consumer preferences and regulatory frameworks ( Isanovic et al., 2023). Adapting strategies to ensure operational resilience and quality standards is crucial for sustained success in the ASEAN consumer goods market.

### Problem statement

ASEAN SMEs in the consumer goods sector face significant supply chain risks that impact their operational resilience. These challenges include limited financial resources, making them vulnerable to disruptions like natural disasters and economic downturns ( Kurniawan et al., 2024). Reliance on a few suppliers exacerbates risks such as insolvency and quality issues ( Panchal et al., 2023). Dynamic markets with fluctuating demand further complicate supply shortages and production bottlenecks ( Annamalah et al., 2018). Additionally, limited technological capabilities hinder supply chain visibility and risk mitigation ( El-Garaihy et al., 2024), while inefficient inventory management and constrained transportation options increase vulnerability ( El Jaouhari & Hamidi, 2024). Regulatory compliance and quality issues compound these risks ( Yang et al., 2024). Furthermore, the lack of in-house expertise and a comprehensive risk management strategy heighten susceptibility ( Derouiche et al., 2021). Insufficient data and analytics capabilities impede accurate demand forecasting ( Nguyen et al., 2023), and difficulties in adopting advanced technologies and navigating complex regulations persist ( Pushp & Ahmed, 2023). Scaling to accommodate growth strains existing processes and infrastructure. Addressing these challenges necessitates effective risk management tools and strategies ( Habbal et al., 2024). However, research combining quantitative and theoretical methods is lacking in ASEAN nations ( Duong et al., 2023), and validated management procedures are needed to mitigate supply chain risks effectively ( Ha & Chuah, 2023). Dynamic consumer behavior and the evolving retail landscape present both opportunities and challenges for consumer goods manufacturers ( Raghavan et al., 2024). To adapt, companies must understand local preferences and compete with modern retail formats ( Mujianto et al., 2023). Robust e-commerce strategies leveraging improved internet access are crucial ( Shanmugalingam et al., 2023). Optimizing the supply chain for efficient logistics and differentiation through innovation is also vital ( Ivanov & Keskin, 2023; Annamalah et al., 2020). Success demands adaptability, strategic planning, and deep market understanding ( Raghavan et al., 2024). This study aims to explore the relationship between supply chain risks and supply chain performance in the consumer products sector within ASEAN nations. Specifically, it investigates whether risk monitoring and risk mitigation serve as mediators in this relationship. The concept of “supply chain performance” in this context refers to the efficiency and effectiveness of the supply chain in meeting end-customer demands, ensuring commodity availability, on-time deliveries, and maintaining sufficient inventory and capacity across the supply network, from raw materials to the final consumer. By clarifying and differentiating these terms, the study seeks to provide a comprehensive understanding of how supply chain risk management practices can enhance performance outcomes among SMEs in the ASEAN consumer product sector.

### Objective

The primary objective of this research is to adopt a comprehensive approach to supply chain risk management to mitigate the impact of supply chain risks and enhance supply chain performance among SMEs entrepreneurs in ASEAN nations that are engaged in consumer product production. In addition, this study aims to explore the interplay between supply chain risks and supply chain performance in the consumer products sector. Specifically, it seeks to investigate whether the monitoring of risks acts as a mediator in the relationship between supply chain risk management and performance, as well as whether risk mitigation similarly mediates the relationship between supply chain risk management practices and performance outcomes among ASEAN nations.

## Literature review

### Supply Chain Performance (SCP)

Supply Chain Performance (SCP) is increasingly recognized as a multidimensional construct that goes beyond operational efficiency to encompass responsiveness, resilience, and strategic alignment with evolving customer expectations. Liu et al. (2023) emphasize that SCP involves meeting end-customer demands through timely deliveries, adequate inventory, and product availability, highlighting its role in ensuring customer satisfaction. However, as Zaman and Kusi-Sarpong (2024) point out, changing socioeconomic dynamics and rising consumer expectations have redefined performance benchmarks, necessitating a shift toward higher service quality and adaptability. A synthesis of recent studies reveals that SCP is shaped by a complex interplay of operational, financial, strategic, and external risks ( Guo et al., 2024). While operational risks may arise from internal disruptions such as production delays or logistics failures, financial and strategic risks are often externally driven such as currency volatility or regulatory changes that demand proactive planning. In contrast, external shocks like natural disasters or global pandemics amplify vulnerabilities across the supply chain ecosystem ( Umar & Wilson, 2024). To navigate these multifaceted risks, scholars converge on the importance of resilience and adaptability. Guntuka et al. (2024) argue that dual sourcing, inventory optimization, and inter-organizational collaboration are foundational to a responsive supply chain. These mechanisms not only buffer against disruption but also enable agility in recovery. Moreover, integrating risk management practices such as continuous monitoring and technology adoption can provide a competitive edge, particularly when combined with sustainability-driven strategies ( Kwaramba et al., 2024). Thus, SCP today is not merely about maintaining flow efficiency, but about fostering resilience, agility, and long-term value creation in an unpredictable global environment.

### Significance of supply risk in the context of supply chain performance

The significance of supply risk in shaping overall supply chain performance (SCP) has gained increasing scholarly attention due to the complex and volatile nature of global operations. Shelar et al. (2023) argue that risk assessment in supply chain management spans across strategic, financial, and operational domains, underscoring its multidisciplinary relevance and the need for integrated approaches. According to Rafi-Ul-Shan et al. (2024), risk is not merely a disruption but a fluctuation in expected outcomes, often resulting from misalignments or shocks within interconnected supply chain components. This interpretation aligns with the reality that local logistics managers and global supply chain leaders perceive and experience risks differently, shaped by both micro-level disturbances and macro-level uncertainties. Supply risks can be internal or external, with natural disasters, geopolitical tensions, and pandemics representing external threats, while supplier reliability and quality represent internal vulnerabilities ( Hajmohammad et al., 2024). What unifies these risk types is their potential to cascade through the supply chain, leading to breakdowns in logistics, production, or customer fulfillment. Studies emphasize supplier failure as a critical risk factor, with multiple sourcing strategies shown to reduce the likelihood of such disruptions ( Hajmohammad et al., 2024). Porter’s value chain model further illustrates that supply risk not only affects procurement and operations but also has downstream implications for outbound logistics and overall competitive performance ( Bustinza et al., 2024). Ensuring robust infrastructure, including affordable access to energy, communication, and digital technology, becomes essential in supporting resilience and productivity in enterprise decision-making. Ngo et al. (2023) highlight that managing supply risk is vital to sustaining high SCP, typically evaluated through efficiency, responsiveness, and effectiveness. Disruptions can erode these metrics, triggering a ripple effect of increased costs, inventory shortages, delivery delays, and reputational damage. To counteract these effects, researchers recommend proactive strategies such as supplier diversification, safety stock management, investment in supply chain visibility technologies, and dynamic risk assessment protocols ( Negri et al., 2024). By synthesizing these insights, it becomes clear that supply risk is not an isolated concern but a central determinant of supply chain agility, resilience, and long-term competitiveness.

Supply risk, as a latent factor, negatively affects the reliability, responsiveness and efficiency of supply chains. High supply risk levels manifested in disruptions, delays, and quality inconsistencies undermine the capacity of supply chains to meet customer expectations and operational targets. Therefore, SCP is hypothesized to be significantly influenced by fluctuations in the latent variable Supply Risk, which captures the breadth and severity of underlying vulnerabilities.

H1a: There exists a significant relationship between supply risk and supply chain performance.

### Significance of supply risk in the context of risk monitoring

Understanding the interplay between supply risk and risk monitoring is essential for maintaining resilient and responsive supply chain operations. Internal risks those within the supplier’s control differ from external risks such as political instability or natural disasters ( Yu et al., 2023; Haraguchi et al., 2023). A comprehensive risk management approach must therefore integrate both risk types, emphasizing not only their identification but also tailored strategies to mitigate their distinct impacts. While some external risks may be unavoidable, such as natural catastrophes, their consequences can be significantly reduced through advance preparation and risk-aware strategic planning ( Haraguchi et al., 2023). The dynamic nature of supply chains establishes a reciprocal relationship between supply risk and risk monitoring, where the former represents potential disruptions and the latter ensures those disruptions are identified, assessed, and managed in real time. This relationship is more than functional, it is strategic, as effective risk monitoring hinges on a deep and evolving understanding of supply risks ( Berger et al., 2023). Thus, monitoring transforms risk management from a reactive function into a proactive and preventive one. The early detection of supplier financial instability through monitoring systems enables companies to respond preemptively by diversifying suppliers or developing contingency plans ( Jessin et al., 2023). This underscores the role of monitoring in enabling agility, not just stability. Given the shifting nature of supply risks arising from market volatility, geopolitical events, or technological disruptions risk monitoring must be continuous and adaptive. This responsiveness allows organizations to revise their mitigation strategies in alignment with changing regulatory and market conditions. Effective monitoring depends heavily on high-quality data, including supply chain performance metrics, external market signals, and predictive analytics, which collectively inform real-time decision-making ( Casagli et al., 2023). Moreover, risk monitoring not only anticipates threats but also evaluates the effectiveness of mitigation strategies, ensuring an iterative feedback loop for improvement. In doing so, it reduces reliance on costly emergency responses and facilitates smoother, more cost-efficient operations ( Pham et al., 2023). Ultimately, integrating supply risk and risk monitoring fosters a culture of vigilance, adaptability, and strategic foresight within supply chain management. These concepts, when effectively aligned, serve as twin pillars safeguarding operational continuity and long-term supply chain resilience.

Risk monitoring represents an organizational process latent construct involving continuous identification, assessment, and tracking of potential risks. The existence of high supply risk (latent) necessitates more robust risk monitoring practices. As organizations perceive greater supply risk, they are more likely to invest in real-time intelligence systems, supplier audits, and contingency tracking mechanisms. Thus, the perception and presence of supply risk drive the depth and frequency of risk monitoring activities, supporting the latent causal link in H1b.

H1b: Supply risk has a significant relationship with risk monitoring.

### Significance of supply risk in the context of risk mitigation

In the consumer products industries, supply risk refers to the possibility of disruptions, uncertainties, or vulnerabilities that may negatively impact the production, distribution, or availability of goods ( Kristanto & Kurniawati, 2023). These risks have become more prominent due to growing dependence on globalized supply networks, which amplify exposure to geopolitical, economic, and regulatory volatility. Companies sourcing raw materials, components, or finished goods across borders face risks such as geopolitical tensions, currency fluctuations, and trade restrictions ( Kristanto & Kurniawati, 2023). To counter these challenges, firms must implement comprehensive and forward-looking mitigation strategies that emphasize resilience, flexibility, and continuity planning. Disruptions such as transportation breakdowns, infrastructure failures, and production halts caused by natural or man-made events highlight the necessity of integrated risk mitigation frameworks that prioritize proactive over reactive measures ( Birge et al., 2023). The complex, interdependent nature of global supply chains in this sector requires continuous monitoring and collaborative risk-sharing across nodes to safeguard operations. Thorough analysis of supplier relationships and network structures allows early identification of vulnerabilities, thereby enabling actions like supplier diversification, dual sourcing, and robust contingency planning ( Berger et al., 2023). Strategic partnerships that foster transparency and trust across the supply network enhance preparedness for unforeseen shocks. Compliance risk also plays a critical role in risk mitigation, especially given the regulatory diversity across ASEAN and international markets. Non-compliance can trigger costly disruptions such as product recalls, halted production, and reputational damage ( Kaewnuratchadasorn, 2023). Hence, compliance management must be treated as a core component of supply risk mitigation, embedded within governance structures and supported by continuous regulatory scanning. Additionally, market-driven uncertainties stemming from fluctuating consumer demand, changing preferences, and emergent trends demand adaptable supply chain strategies rooted in real-time intelligence and agile operations. Forecasting tools and market analytics can enable firms to respond swiftly to shifts in demand patterns. The COVID-19 pandemic underscored the fragility of supply chains, particularly in ASEAN, by exposing vulnerabilities tied to just-in-time inventory systems and overreliance on single-source suppliers ( Todo et al., 2023). This calls for a reevaluation of lean strategies in favor of buffers that enhance resilience. Moreover, the digital transformation of supply chains introduces cybersecurity as an emerging dimension of supply risk. Attacks targeting suppliers can disrupt operations, compromise sensitive information, and cascade across the supply network. Therefore, cybersecurity readiness including vendor audits, secure data systems, and incident response protocols must be integral to any comprehensive risk mitigation approach. In sum, effective risk mitigation in the consumer products sector requires a holistic strategy that integrates supplier diversification, regulatory compliance, agile demand management, and digital security. Such integration enhances the robustness and adaptability of supply chains, enabling firms to sustain performance and competitiveness amidst ongoing uncertainty.

Risk mitigation is also a latent construct reflecting strategic and tactical actions aimed at reducing or controlling the impact of supply-related disruptions. These include supplier diversification, inventory buffers, nearshoring, or regulatory compliance strategies. When supply risk is high as measured by multiple latent indicators as organizations are more compelled to proactively initiate mitigation measures. Therefore, supply risk acts as an antecedent latent variable that triggers enhanced investment in mitigation capabilities.

H1c: Supply risk has a significant relationship with risk mitigation.

### Significance of process risk in the context of supply chain performance

Process risk is a critical component of supply chain management, bearing substantial consequences for operational efficiency, product quality, and customer satisfaction ( Prime et al., 2020). It encompasses internal disruptions within a company’s operations such as equipment failures, labor shortages, or quality deviations that may interrupt the seamless flow of products and services ( Shekarian & Mellat Parast, 2021). These disruptions, though internal, often create ripple effects across the broader supply chain network, making process risk a vital focal point for supply chain resilience. Machine breakdowns, production errors, or worker strikes can severely constrain manufacturing throughput, delay deliveries, and reduce service levels ( Shekarian & Mellat Parast, 2021). The inherent unpredictability in production systems such as variability in machine availability or process cycles introduces uncertainty and complicates the maintenance of consistent output levels ( Park et al., 2024). This variability adversely impacts key performance indicators like throughput time, capacity utilization, process yield, and product conformity, creating inefficiencies and bottlenecks in operations. Prolonged throughput times can delay customer deliveries, while quality inconsistencies can result in product rework, scrap, or warranty claims all of which erode supply chain performance and customer trust ( Daultani et al., 2024). As a result, process risk management becomes an essential enabler of operational excellence, especially in environments where time-to-market and quality consistency are strategic priorities. Given that efficient supply chain performance is heavily reliant on reliable internal processes, firms must actively monitor, assess, and mitigate process risks. Proactive measures such as predictive maintenance, employee training, total quality management (TQM), and process automation play a pivotal role in reducing variability and enhancing process reliability. Firms can also employ real-time monitoring systems and lean manufacturing principles to detect early warning signs of process deterioration and swiftly implement corrective actions ( Munir et al., 2024). Despite its strategic significance, process risk often receives less attention compared to more visible risks like demand uncertainty or supplier disruptions. This oversight can lead to gaps in risk mitigation frameworks, particularly in high-volume or continuous production systems where even minor process inefficiencies can escalate into major operational setbacks. Therefore, future research should delve deeper into process risk typologies, interdependencies with other supply chain risks, and the design of adaptive mitigation strategies to strengthen supply chain robustness ( Liu et al., 2024). In sum, effectively managing process risk is essential for maintaining the consistency, speed, and quality of supply chain outputs. Addressing this often-underestimated risk area enables firms to improve not only their internal operations but also their capacity to meet external market demands reliably.

Process Risk is treated as a latent variable that encompasses various internal disruptions within organizational processes. These disruptions, such as equipment failures, quality control issues, and operational delays, are not directly observable but can be inferred through measurable indicators. Recognizing process risk as a latent construct enables a holistic understanding of how these internal inefficiencies impact the broader supply chain, particularly in terms of throughput time, service reliability, and customer satisfaction. This approach provides a robust foundation for examining its relationship with supply chain performance.

H2a: Process Risk has a significant relationship with Supply Chain Performance.

### Significance of process risk in the context of risk monitoring

The significance of process risk within the domain of risk monitoring is an indispensable facet of contemporary business operations, as process risk refers to the potential disruptions, errors, or deviations that may manifest within an organization’s operational workflows ( Tsang et al., 2018). Effective risk monitoring serves as the linchpin for identifying, evaluating, and mitigating these risks, thereby ensuring the seamless functionality of business processes and the achievement of organizational objectives ( Pham et al., 2023). A comprehensive approach to risk monitoring starts with the identification of potential risks, which arise from both internal factors, such as complex process design, resource constraints, and insufficient employee training, as well as external factors, including regulatory changes, market fluctuations, and technological shifts. This identification process is fundamental, as recognized risks must undergo thorough assessment and quantification, involving continuous evaluation to gauge their likelihood and potential impact on operational processes. Real-time data analytics, performance metrics, and key performance indicators (KPIs) are crucial tools in this ongoing process, providing actionable insights into the risk landscape ( Aljohani, 2023). However, risk monitoring goes beyond mere assessment, extending into the development and implementation of strategic mitigation actions, such as process redesign, technological upgrades, and employee capacity building. Additionally, ensuring compliance with relevant regulatory standards becomes a core aspect of risk mitigation, as failure to comply could expose businesses to legal liabilities and financial penalties. The role of process risk in risk monitoring is pivotal, as it underpins the dependability and effectiveness of organizational processes. By integrating continuous risk evaluation, quantification, and proactive mitigation into daily operations, businesses can enhance their resilience against both anticipated and unforeseen disruptions. A process entails crucial management functions within an organization, relying heavily on locally owned resources and operational infrastructure, which are susceptible to disruptions that affect product flow, such as equipment failures, technological advances, labor disputes, and quality control issues ( Mc Loughlin et al., 2023). Moreover, the inherent unpredictability of manufacturing systems such as machine downtime, worker availability, and fluctuations in process flow can negatively impact key metrics like throughput time, yield rates, and product quality. Process risk threatens a producer’s ability to fulfill customer orders promptly, compromising both operational efficiency and supply chain performance. Despite the increasing attention given to demand and supply-side risks, it is imperative that more research is devoted to process risk, particularly in the context of risk monitoring systems that can identify, measure, and mitigate these risks early on (Pham et al., 2023). Addressing process risk is not only about mitigating operational disruptions but also about ensuring a sustainable and responsive supply chain capable of adapting to dynamic conditions.

Process Risk is conceptualized as a latent construct consisting of internal variabilities and potential disruptions in workflow execution. These risks such as unexpected machine downtime, labor inconsistencies, and deviations in standard operating procedures cannot be directly measured but are reflected in operational data and key performance indicators. Modeling process risk as a latent variable allows for a nuanced understanding of how organizations monitor, detect, and respond to emerging threats, thus reinforcing the importance of systematic surveillance and proactive risk identification mechanisms.

H2b: Process risk has a significant relationship with risk monitoring.

### Significance of process risk in the context of risk mitigation

The significance of process risk within the context of risk mitigation is crucial to contemporary business operations. Process risk refers to potential disruptions, errors, or deviations within an organization’s operational workflows, making effective management essential for identifying, assessing, and mitigating these risks ( Schuett, 2023). This proactive management ensures the smooth functioning of business processes and the achievement of organizational goals. Identifying process risks requires considering a wide range of sources, including internal factors such as process intricacies, resource constraints, and employee errors, alongside external factors like regulatory changes and supply chain disruptions. Methodologies for identifying operational risks, which often overlap with process risks, are critical for organizations to systematically pinpoint vulnerabilities and failure points within their operations. This systematic approach allows for proactive risk mitigation, ensuring the continuity and resilience of business processes ( Fang et al., 2024). To effectively manage these risks, it is necessary to quantify and assess their potential impact and likelihood. This comprehensive evaluation provides a clear understanding of their consequences and probability of occurrence. Quantitative methodologies such as risk matrices and Monte Carlo simulations are commonly employed to evaluate and quantify process risks. These tools offer structured frameworks for assessing both the severity and probability of operational risks, ultimately enhancing decision-making and strengthening risk management strategies ( Tunçel et al., 2023). Continuous monitoring, supported by real-time data, key performance indicators (KPIs), and performance metrics, is essential to detect deviations and emerging risks as they arise. Data analysis techniques for continuous monitoring are invaluable for identifying trends, anomalies, and potential risks, allowing for timely and proactive risk mitigation measures that safeguard operations and prevent disruptions. Risk monitoring extends beyond observation, incorporating the implementation of strategies such as process redesign, technological improvements, training programs, and contingency planning ( Menz et al., 2024). In addition, stringent regulatory requirements, particularly in industries such as finance, further highlight the importance of mitigating process risk to ensure compliance and avoid legal consequences. In summary, process risk plays a central role in risk mitigation, ensuring the reliability and efficiency of organizational processes. By continuously identifying, assessing, and addressing these risks, organizations can maintain a resilient and agile operational framework.

Process Risk is treated as a latent variable inferred from observable indicators like process delays, quality defects, system breakdowns, and operational inefficiencies. These underlying risks are multifaceted and may not be directly visible until their impact is manifested. Framing process risk as a latent construct supports a comprehensive evaluation of mitigation strategies, such as preventive maintenance, training programs, and process redesign, all aimed at minimizing disruption and enhancing process resilience.

H2c: Process risk has a significant relationship with risk mitigation.

### Significance of demand risk in the context of supply chain performance

Demand risk plays a crucial role in influencing supply chain performance, particularly for Small and Medium-sized Enterprises (SMEs). Due to their size, limited resources, and constrained capabilities, SMEs are highly vulnerable to demand fluctuations, making them more susceptible to inaccuracies in demand forecasting. These inaccuracies can result in overstocking or stockouts, which not only increase carrying costs but also lead to missed sales opportunities, ultimately affecting the financial stability of SMEs ( Ali et al., 2023; Gurbuz et al., 2023). The unpredictability of demand further strains the financial health of SMEs by impeding their ability to manage operational expenses and invest in key business areas ( Patil & Prabhu, 2024). Moreover, the heavy dependence of SMEs on a limited number of suppliers exacerbates the impact of demand variability, potentially jeopardizing supplier relationships and disrupting the flow of critical production inputs ( Deng et al., 2022). In turn, inconsistent product availability resulting from demand uncertainties can negatively affect customer satisfaction, ultimately harming the brand image and eroding customer loyalty ( Dewi & Hastuti, 2024). The lack of flexibility within SMEs to rapidly adapt to demand fluctuations complicates their ability to adjust production schedules and scale operations accordingly ( AlZayani et al., 2024). However, investing in affordable, user-friendly technology solutions could provide SMEs with the necessary tools to better understand demand patterns and respond to changes more effectively ( AlZayani et al., 2024). In light of these challenges, it is vital for SMEs to recognize the significance of demand risk in their supply chain management. Developing resilient and agile supply chains that can effectively navigate demand uncertainties is key to sustaining growth and remaining competitive in an ever-evolving market ( Lu et al., 2024). Managing demand risk effectively is essential for improving various aspects of supply chain performance, including financial stability, supplier relations, customer satisfaction, and operational adaptability. To excel in managing these risks, SMEs must adopt robust demand forecasting methods and risk management strategies ( Lu et al., 2024).

Demand Risk is conceptualized as a latent variable that captures the uncertainty and volatility in customer demand, which cannot be directly observed but is inferred through related indicators such as sales variability, forecast errors, and market fluctuations. This construct encompasses hidden patterns and unpredictable shifts in customer preferences, economic changes, and promotional effects that can significantly influence inventory levels, order fulfillment, and financial performance. Treating demand risk as a latent variable enables researchers to model its indirect but substantial impact on supply chain outcomes, especially for SMEs that are particularly vulnerable to demand disruptions.

H3a: Demand risk has a significant relationship with supply chain performance.

### Significance of demand risk in the context of risk monitoring

Demand risk has become a critical component in risk monitoring frameworks, especially in dynamic and volatile markets ( Tantsyura et al., 2016). It refers to the unpredictability of customer demand, which significantly influences a company’s revenue, production capabilities, and overall performance ( Thi Binh An et al., 2024). As such, effective demand risk management is vital for businesses striving to remain adaptable, competitive, and financially stable ( Fu et al., 2023). This risk encompasses a variety of factors, including economic fluctuations, changes in consumer preferences, competitive dynamics, and external influences such as natural disasters or pandemics. Demand risk directly impacts key elements of supply chain management, such as inventory management, production planning, and operational efficiency. When demand is misjudged, it can lead to severe consequences overestimating demand results in excess inventory and associated carrying costs, while underestimating demand leads to stockouts and missed sales opportunities ( Zhu et al., 2024). To mitigate demand risk, companies must strike a balance between inventory levels, production capacities, and workforce management to ensure they can respond effectively to demand fluctuations. A resilient supply chain is crucial to adapting to these demand oscillations and ensuring consistent product availability ( Swaminathan & Venkitasubramony, 2024). Modern demand forecasting models and advanced analytics are invaluable tools for accurately predicting demand patterns, enabling businesses to make proactive adjustments to mitigate risks ( Khan et al., 2020). Demand risk becomes especially pronounced in industries with high volatility, triggered by factors like changes in order volumes, shorter product life cycles, or the introduction of new products. In consumer goods supply chains, demand risk can have long-term implications, such as unforeseen shifts in consumer demand or adjustments to food safety standards ( Prashar & Vijaya, 2024). Thus, managing demand-related risks is essential for ensuring strong supply chain performance, particularly in sectors like consumer products. Contemporary academic research offers valuable insights into best practices for managing demand risk and its broader implications for business sustainability and performance ( Hasan et al., 2024). This highlights the critical importance of understanding and mitigating demand-related risks within risk monitoring strategies for enhancing supply chain resilience.

Demand Risk is viewed as a latent construct comprising unpredictable changes in market needs and consumer behavior. While the specific causes of demand shifts such as competitive actions or global events may not be directly measurable, their effects are observable through changes in sales trends, customer orders, and inventory fluctuations. Recognizing demand risk as a latent variable enables the systematic capture of these unobservable variations and supports the development of effective monitoring systems. These systems help firms anticipate potential demand disruptions and adapt their operations, accordingly, thereby improving responsiveness and preparedness.

H3b: Demand risk has a significant relationship with risk monitoring.

### Significance of demand risk in the context of risk mitigation

Demand risk plays a crucial role in risk mitigation strategies, particularly in the context of modern business operations, where unpredictable market conditions and evolving consumer behaviors pose substantial challenges. This risk pertains to the variability and unpredictability of customer demand for products or services, which can have a profound impact on revenue, production schedules, inventory management, and overall business performance ( Khan et al., 2024). Effective demand risk management is vital for businesses, as it enables them to allocate resources more efficiently, maintain operational flexibility, and ensure financial stability in the face of uncertainty. Demand risk can arise from several sources, including economic fluctuations, changes in consumer preferences, competitive pressures, and unforeseen disruptions, such as supply chain interruptions or global crises ( Ngo et al., 2024). The financial consequences of demand risk can be significant. Overestimating demand may lead to excessive inventory levels, which in turn incur high holding and storage costs, while underestimating demand may result in lost sales opportunities and customer dissatisfaction ( Annamalah et al., 2018). Balancing inventory levels with demand variability is a key aspect of managing this risk and is essential for building supply chain resilience ( Rohaninejad et al., 2023). A well-balanced inventory system enables companies to maintain product availability while minimizing excess stock, making it crucial for fostering operational stability. This relationship between demand risk and supply chain resilience highlights the importance of achieving equilibrium between supply and demand. A resilient supply chain is not only responsive to fluctuations in demand but also ensures that products are consistently available, minimizing disruptions and maximizing customer satisfaction. To mitigate demand risk, businesses must employ robust demand forecasting models and advanced analytics, which allow for more accurate predictions of future demand patterns ( Swaminathan & Venkitasubramony, 2024). These tools empower companies to make proactive decisions regarding production planning, resource allocation, and inventory management, reducing the risks associated with demand uncertainty. Moreover, staying vigilant to market dynamics, consumer behavior trends, and competitive pressures is essential for effectively managing demand risk. By continuously monitoring these factors, businesses can adjust their strategies to remain agile and responsive to shifts in demand, thus maintaining a competitive edge in an ever-changing market environment.

Demand Risk is understood as a latent variable characterized by variability in customer requirements and unpredictable market dynamics. Though the precise triggers such as evolving consumer tastes or external shocks that are not directly visible, their influence manifests through fluctuating demand signals and inconsistent sales patterns. Modeling demand risk as a latent construct allows for a deeper exploration of how firms implement mitigation strategies such as flexible production systems, demand-driven inventory practices, and enhanced forecasting tools. This conceptualization supports the proactive management of risks and helps maintain supply chain continuity amid uncertainty.

H3c: Demand risk has a significant relationship with risk mitigation.

### Significance of risk monitoring in the context of supply chain performances

In modern business environments, risk monitoring is a vital element for ensuring the performance and resilience of supply chains, especially given the complexity and interconnectedness of global supply networks ( Yang et al., 2023). It involves continuous assessment and proactive mitigation of potential risks that could disrupt supply chain operations, thereby impacting cost efficiency, product quality, and customer satisfaction. By continuously monitoring risks, organizations can identify potential threats such as natural disasters, geopolitical tensions, supplier disruptions, or cyberattacks ( Ganesh & Kalpana, 2022), allowing them to implement preemptive strategies to minimize their impact. Supply chain disruptions, whether through operational downtime, late deliveries, or inventory shortages, can have far-reaching effects on customer satisfaction and brand reputation. Risk monitoring plays a crucial role in maintaining operational continuity by ensuring that emerging risks are quickly addressed. This allows for optimized resource allocation and cost-efficiency improvements ( Mohezar et al., 2023). By identifying potential disruptions early on, organizations can avoid the additional expenses associated with emergency orders, excessive inventory holdings, or delays in production. This proactive approach contributes directly to operational stability, ensuring that supply chains remain agile and responsive to market demands. Moreover, continuous risk monitoring fosters stronger, more transparent relationships with suppliers by enabling joint risk mitigation strategies. This collaboration enhances the resilience of the entire supply chain, improving its ability to weather uncertainties and reduce vulnerabilities ( Hajmohammad et al., 2024). Additionally, monitoring the regulatory landscape is essential, as shifts in laws and compliance standards can pose significant risks to supply chain operations. Risk monitoring helps organizations navigate these changes, minimizing legal and financial consequences ( Bangar et al., 2024). Ultimately, effective risk monitoring leads to improved customer satisfaction. By ensuring that supply chains are agile and efficient, organizations can consistently deliver products and services on time, meeting or exceeding customer expectations. Regularly assessing and mitigating risks ensures that the supply chain operates smoothly, even in the face of unforeseen challenges, thereby optimizing overall performance ( Wiryasaputra et al., 2023).

Risk Monitoring is conceptualized as a latent variable in the context of supply chain performance, representing the continuous and often hidden processes involved in identifying, evaluating, and responding to risks. While direct observation of all potential risks such as geopolitical threats, supplier failures, or cyber-attacks may not be feasible, their effects can be seen in disruptions and performance variances within the supply chain. Modeling Risk Monitoring as a latent construct helps capture the underlying dynamic of risk detection and response, which is central to mitigating supply chain disruptions and optimizing operational efficiency. The effectiveness of risk monitoring is indirectly inferred through indicators like response time to disruptions, resource reallocation, and improved communication among supply chain partners. Understanding it as a latent variable allows businesses to better conceptualize how continuous risk evaluation supports supply chain resilience and enhances customer satisfaction by ensuring smooth and timely operations.

H4: Risk monitoring significantly mediates the relationship between the factors and supply chain performance

### Significance of risk mitigation in the context of supply chain performances

In contemporary business environments, risk mitigation is crucial for ensuring optimal supply chain performance, particularly in a world characterized by global interconnectivity and rising complexities ( Dohale et al., 2023). It involves developing and implementing strategies to identify, assess, and mitigate risks, thereby ensuring the smooth flow of goods, cost-effectiveness, and customer satisfaction ( Hashim et al., 2024). Effective risk mitigation begins with the systematic identification and evaluation of potential risks within the supply chain, such as disruptions, supplier issues, and external threats ( Wiryasaputra et al., 2023). This process is essential for crafting effective strategies to mitigate risks and prevent them from affecting the operations. Risk mitigation goes beyond simply identifying risks as it also ensures business continuity by reducing the impact of disruptions. This is achieved through the development of contingency plans, the establishment of alternative suppliers, and the implementation of quick response strategies ( Dohmen et al., 2023). Such measures contribute significantly to cost efficiency by preventing unnecessary expenses that might arise from disruptions, such as urgent orders or holding excessive inventory ( Groenewald et al., 2024). By addressing risks proactively, organizations enhance supply chain resilience, enabling rapid recovery and adaptation when faced with challenges ( Alsmairat, 2023). Additionally, risk mitigation fosters stronger relationships with suppliers through transparent communication and collaborative efforts to manage shared risks. This collaborative approach enhances the resilience of the entire supply chain network ( Alsmairat, 2023). Furthermore, risk mitigation directly influences customer satisfaction, as a resilient supply chain ensures the timely delivery of products and services, which in turn strengthens customer loyalty and trust.

The effectiveness of risk mitigation is also contingent upon the insights gained from comprehensive risk assessments. A thorough understanding of potential risks is essential for devising appropriate mitigation strategies ( Hochrainer-Stigler et al., 2024). In consumer product supply chains, where entrepreneurs often face high risks but may have limited awareness, sharing risk information becomes critical for minimizing risks and fostering long-term, sustainable performance ( Muttitt et al., 2023). This research highlights the importance of knowledge-sharing in enhancing the effectiveness of risk mitigation practices, which is key to achieving sustainable supply chain performance.

Risk Mitigation is treated as a latent variable that involves the proactive strategies organizations use to identify, assess, and reduce supply chain risks. While the individual actions, such as developing contingency plans or securing alternative suppliers, are observable, the underlying process of risk reduction itself cannot always be directly measured. By conceptualizing risk mitigation as a latent variable, we can capture the hidden but essential mechanisms that help organizations manage uncertainties and maintain smooth operations despite disruptions. This variable encompasses strategies such as inventory optimization, alternative sourcing, and rapid response measures, which contribute indirectly to improving supply chain performance. Understanding the latent nature of risk mitigation enables firms to focus on the dynamic interplay between their strategies and how these measures affect operational efficiency, cost-effectiveness, and, ultimately, customer satisfaction.

H5: Risk mitigation significantly mediates the relationship between the factors and supply chain performance.

### Underpinning theory

**Figure 1.**
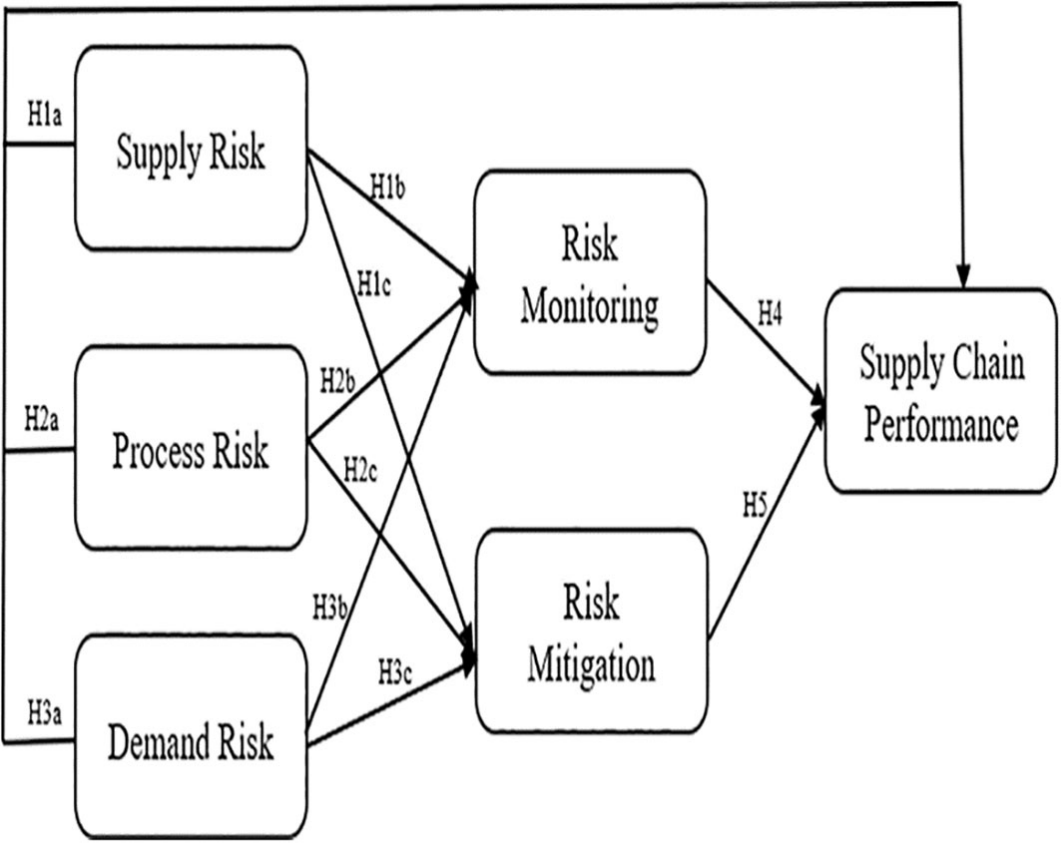
Research Framework of the Impact of Risk Variables on Supply Chain Performance Mediated by Risk Monitoring and Mitigation. Modified from various studies: Aqlan & Lam (2015); Ho et al. (2015); Kern et al. (2012).

The purpose of risk identification is fundamental to supply chain management, aiming to uncover all relevant risks and anticipate future uncertainties for proactive management. Without identifying risks, no risk management measures can be implemented. Early assessment during risk identification is crucial to determine the relevance of a risk and whether further assessment or mitigation is necessary ( Cevikbas et al., 2024). A comprehensive approach to risk identification is essential to identify all potential threats and vulnerabilities in the supply chain. Successful Supply Chain Risk Management (SCRM) requires a thorough yet swift and cost-effective evaluation of Supply Chain Risks (SCRs), which can be achieved through risk assessment utilizing available data or expert judgment and scenarios ( Chen et al., 2024). Integrating objective data with subjective perceptions enhances the robustness of risk identification, improving the accuracy of risk prediction and assessment. Prioritizing risks is crucial for organizations to focus on the most significant ones. Risks with substantial impact or those that can be promptly mitigated are assigned high priority ( Gupta et al., 2023). Risk prioritization aids in determining which risks to target with actions, enabling efficient allocation of limited resources for risk treatment ( Annamalah et al., 2019). Understanding the interconnections among risks and their cascading effects is vital for prioritizing risks, devising treatment plans, and executing effective risk management measures ( Schulman, 2023). Identifying the most crucial risk capable of triggering multiple risks is essential for effective risk management. Continuous monitoring is necessary to ensure that acknowledged risk consequences do not intensify. If consequences surpass a specified threshold, organizations must contemplate strategies to evade, transfer, share, or mitigate the risk ( Habbal et al., 2024). Mitigation aims to decrease risk to an acceptable level, addressing both the likelihood and consequences of a risk event. The choice of a mitigation strategy is influenced by the risk type, organization’s budget, and careful evaluation of acceptance, avoidance, sharing, and transfer options ( Gurbuz et al., 2023). Given the interconnected nature of risks, mitigating one type may exacerbate or alleviate another. Therefore, mitigation strategies should be applied with minimal contradictions and careful consideration of risks with negative dependencies. Investing in risk avoidance becomes essential for high probability, high impact risks, while risk acceptance may be considered for low probability, low impact risks ( Aldrighetti et al., 2023). Risk is dynamic and requires ongoing monitoring to assess the evolution of risk sources and determine whether adjustments to treatment strategies are necessary. Effective Supply Chain Risk Management (SCRM) contributes to improved supply chain performance by enhancing resilience, reducing disruptions, and ultimately improving overall performance ( Emrouznejad et al., 2023). Continuous risk monitoring, proactive risk mitigation, and effective response planning are crucial elements in achieving this objective. Figure 1 presents the research framework that examines the impact of various risk variables on supply chain performance, with a particular focus on the mediating role of risk monitoring and mitigation.

## Methods

### Construct Development and Item Selection

The constructs for risk monitoring, risk mitigation, and supply chain resilience were developed by adapting existing, well-established models in the supply chain risk management literature. The risk monitoring construct is based on the framework of Khan et al. (2024), which identifies key elements such as continuous assessment of risk exposure, early detection systems, and responsiveness to emerging threats. The risk mitigation construct was adapted from Rohaninejad et al. (2023), incorporating elements that address both proactive and reactive strategies to reduce the impact of identified risks. Similarly, the supply chain resilience construct was grounded in the work of Swaminathan & Venkitasubramony (2024), focusing on a firm’s ability to recover from disruptions, adapt to changing conditions, and maintain service levels during supply chain disturbances. For the item selection, we drew from these validated frameworks, adapting them to the context of SMEs in ASEAN countries, where risk exposure and mitigation strategies may differ due to regional variances in infrastructure, market maturity, and policy environments. Items were carefully selected to align with theoretical definitions while ensuring relevance to the diverse supply chain contexts across ASEAN.

### Research Design and Data Collection

This study follows a deductive research approach and operates within a positivist paradigm, utilizing quantitative methods. Deductive research begins with a broad premise and draws conclusions based on observations and empirical verification ( de Mast et al., 2023). The positivist orientation of this research is evident in its use of quantitative techniques, including surveys and structured questionnaires. To quantify correlations among variables of interest, hypotheses are formulated and rigorously assessed through statistical analysis ( Strunk, 2023). The study concentrates on individuals holding significant authority within SMEs, such as owners, executives, and managers. Primary data is collected through surveys targeted at SME owners and managers across various companies. The questionnaires are administered using a Likert scale format. Confirmatory factor analysis is employed to examine and measure the identified factors, and the study utilizes Partial Least Square Structural Equation Modeling (PLS-SEM) for assessing internal consistency ( Hair et al., 2023). In this study, a probability-sampling method, specifically simple random sampling, was utilized for the selection of SMEs. Careful endeavours were undertaken to maximize the identification of eligible respondents within the consumer product sector, comprising managers, proprietors, and senior executives. In Southeast Asia, as of 2020 there are approximately 71 million micro, small, and medium-sized enterprises (SMEs) ( ASEAN, 2022), constituting 97% of all businesses in the region. These enterprises collectively employ 67% of the working population ( ASEAN, 2022). As of 2022, Southeast Asia is home to a significant number of Micro, Small, and Medium Enterprises (SMEs), which play a crucial role in the region’s economy. SMEs are vital contributors to economic growth, job creation, innovation, and poverty alleviation in Southeast Asian countries. Indonesia has one of the largest populations and economies in Southeast Asia, and it also hosts a substantial number of SMEs. Thailand is another significant hub for SMEs in Southeast Asia. Vietnam has experienced rapid economic growth in recent years, driven in part by the proliferation of SMEs ( ASEAN, 2022). Table 1 presents the data on the quantity of SMEs across ASEAN member states.

**Table 1.** Number of SMEs in Southeast Asia.

Countries	No of SMEs
Indonesia	864,000
Thailand (including Mico) *	3100000
Malaysia (including Mico) *	1101725
Philippines (including Mico) *	1240000
Vietnam (including Micro) *	1109684
Cambodia (including Micro) *	751000
Singapore	309,000
Laos (including Micro) *	126717
Myanmar	126237
Brunei (including Micro) *	6,411

* Data not available separately for SMEs, Total MSMEs (approximately).

Source: ASEAN (2022).

### Ethical statement

We, the undersigned co-authors, affirm that our manuscript titled “Resilience in the Face of Uncertainty: Navigating Supply Chain Challenges Through Proactive Risk Surveillance and Mitigation Strategies among SMEs in ASEAN Countries” contains original material that has not been published, either in full or in part, in any other journal or publication. This manuscript is not under consideration for publication elsewhere. Each author has made significant and active contributions to the research and preparation of this manuscript and accepts joint and individual responsibility for its content. This study was conducted in accordance with the ethical principles outlined in the Declaration of Helsinki. Ethical approval for the research was obtained from the Institutional Review Board (IRB) of SEGi University with the approval number IRB-2023-14532, granted on May 5, 2023. Participants were provided with comprehensive information about the study, including its purpose, procedures, potential risks, and benefits. Written informed consent was obtained from all participants prior to their inclusion in the study. They were assured that their participation was voluntary and that they could withdraw at any time without any consequences. In cases where verbal consent was obtained due to [specific reasons, e.g., participants’ literacy levels, cultural considerations], this approach was approved by the IRB, ensuring that all participants received the necessary information and gave their informed consent voluntarily. The IRB reference number for this approval is IRB-2024-12346.

While this study presents regional-level insights, it recognizes the diverse economic, cultural, and policy contexts across ASEAN countries. Our sample was carefully stratified, as reflected in Table 2, based on country-specific data on SME prevalence and economic activity ( ADB, 2022). During data analysis, we segmented responses by country, allowing us to identify key differences in industry focus and resource access. Singaporean SMEs were more likely to be engaged in digital innovation and technology-driven sectors, whereas SMEs in Cambodia, Laos, and Myanmar operated in traditional sectors and faced infrastructural limitations. Although a full statistical comparison between countries was not within the scope of this study, such distinctions were considered during interpretation of results and are highlighted in our discussion. Future studies should build upon this work by conducting comparative or multilevel analyses to better understand how national factors influence SME development trajectories within the ASEAN region.

**Table 2.** Country respondents.

Countries	No of respondents
Indonesia	94
Thailand	77
Malaysia	63
Philippines	51
Vietnam	25
Singapore	22
Laos	18
Myanmar (Burma)	13
Brunei	12
Cambodia	10
Total	385

The survey sample comprised 385 organizations spanning diverse industries across the surveyed region. These organizations were selected based on statistics provided by ADB (2022) to ensure representation from various business sectors. The objective of the survey is to gather information and insights about different industries, and the selection process aimed to include a representative sample of organizations or businesses from these sectors. The organizations were categorized into specific consumer products sectors according to their primary focus and offerings, which included Technology and Electronics, Fashion and Apparel, Beauty and Personal Care, Health and Wellness, Food and Beverage, among others (refer to Table 3).

**Table 3.** Sample categories of Companies.

Company Type	Organisations samples
Technology and Electronics	33
Fashion and Apparel	32
Beauty and Personal Care	30
Health and Wellness	29
Food and Beverage	27
Home and Kitchen Appliances	26
Toys and Games	25
Furniture and Home Decor	22
Pet Products	21
Sports and Outdoor Equipment	19
Baby and Childcare Products	19
Automotive Accessories	18
Travel and Luggage	18
Consumer Electronics Accessories	16
Jewellery and Accessories	16
DIY and Home Improvement	12
Stationery and Office Supplies	11
Crafts and Hobbies	11
Total	385

Upon developing the survey instrument, a preliminary evaluation was undertaken to assess its suitability and relevance. This pre-test was conducted to confirm that the questionnaire was clear and acceptable to its target audience before its implementation in the actual study, a crucial aspect of an invention survey ( Brzoska et al., 2022). To accomplish this, the questionnaire underwent systematic testing involving participation from managers, owners, and executives holding decision-making positions in SMEs, along with academic researchers. A total of 10 respondents, all of whom were managers, owners, or executives in SMEs within the business industry, were engaged in pre-test to evaluate the questionnaire’s content, readability, and completion time. Subsequently, a pilot study was conducted, involving 40 respondents, which represented approximately 10% of the anticipated sample size for the main study ( Morin, 2023). These respondents included managers, proprietors, and executives actively engaged in the operations of the various businesses. It is noteworthy that, due to issues related to non-responses to specific questions, only 385 out of the initially targeted 500 organizations yielded usable data. The response of the sample size 77 percent (see Table 4).

**Table 4.** Response from respondents.

Response	Instruments
Questionnaires distributed	500
Questionnaires returned	412
Questionnaires excluded	27
Questionnaires useable	385
Response rate	77%

Source: Estimate based on survey data.

### Reliability and validity

To ensure the precision and accuracy of the measurement tools, a reliability assessment was conducted using Cronbach’s Alpha. This assessment plays a crucial role in improving the consistency of measurements by identifying and addressing potential errors. In assessing questionnaire validity, a combination of adopted and adapted items was utilized, with input from academic experts to ensure content validity. Based on the positive feedback from the pilot study and the strong reliability of most scales employed (as depicted in Table 4), only minor adjustments were made to the questionnaire. Table 5 demonstrates that all variables in the study achieved Cronbach’s alpha values exceeding the 0.70 threshold, confirming the robustness of the measurement instruments utilized in this research. The Smart-PLS technique, employing PLS-SEM for data analysis, was utilized to affirm the reliability of the instrument for future administration to actual respondents.

**Table 5.** Reliability assessment of variables.

No	Variable	No. of Items	Cronbach’s α
1	Supply Risk	5	0.812
2	Process Risk	5	0.768
3	Demand Risk	5	0.839
4	Risk Monitoring	4	0.538
5	Risk Mitigation	4	0.786
7	Supply Chain Performance	5	0.822
8	All Items (Total)	28	0.831

Source: Hair et al. (2012).

### Population

SMEs are vital contributors to the economic progress and advancement of ASEAN Member States. With approximately 71 million SMEs in the region, they represent a significant portion, ranging between 97.2% and 99.9% of total establishments within ASEAN Member States. These statistics underscore the pivotal role SMEs play in fostering economic and social development, as they actively engage in value-added activities, drive innovation, and promote inclusive growth by generating employment opportunities and maintaining widespread presence across various sectors ( Annamalah et al., 2016). Therefore, SMEs serve as the cornerstone of ASEAN’s economy, playing an essential role in fostering long-term and sustainable economic growth while also aiding in bridging the development divide.

### Sampling design

In this study, probability sampling, specifically the simple random sampling technique, was employed to select participants. This method was chosen for its practicality in obtaining a comprehensive list of entrepreneurs from SMEs in the consumer goods industry across ASEAN countries. By applying simple random sampling, every entrepreneur within the defined population had an equal chance of being selected, ensuring a representative and unbiased sample. This technique allows for generalization of the findings to the broader population of entrepreneurs in the ASEAN region.

## Analysis

### Common Method Variance (CMV)

Common method variance (CMV) is crucially assessed in self-reported questionnaires, especially when one person provides both predictor and criterion variables. The Harman single factor test is commonly used for this purpose, where each construct undergoes major component factor analysis. If one factor dominates covariance or one general component emerges, CMV exists. This method is essential due to CMV’s significant risk and infrequent replication studies. In this study, the first component accounted for 26% of variance, with no common factor identified, indicating no significant CMV ( Podsakoff et al., 2003). Eigenvalues and Extraction Sums of Squared Loadings provide valuable insights into the effectiveness of the factor extraction process in capturing and explaining the variance in the dataset. The one-factor analysis according to Harman’s method was conducted. The un-rotated factor analysis revealed that the initial factor accounted for only 26.71% of the variance. Consequently, it was concluded that the common method bias did not pose a significant threat in the study, as this percentage falls below the 50% threshold of variance. Table 6 presents the findings of the Harman one-factor analysis for this study.

**Table 6.** Total variance explained.

Component	Initial Eigenvalues			Extraction sums of squared loadings		
	Total	% of Variance	Cumulative %	Total	% of Variance	Cumulative %
1	12.21	26.712	26.712	12.21	26.712	26.712
2	3.589	11.965	5.664			
3	2.558	8.526	6.193			
4	1.999	6.665	6.855			
5	1.309	4.365	7.219			

### Descriptive analysis

Table 7 displays the data’s descriptive statistics as well as the variables’ reasons. The items’ standard deviations varied from 0.63 to 0.92, while the mean ranged from 3.84 to 4.25. The mean and standard deviation figures were within an acceptable range, indicating that most participants agreed with the claims.

**Table 7.** Descriptive statistics (n=385).

Constructs	Mean	Std. Deviation
Supply Risk	4.25	0.63
Process Risk	4.08	0.61
Demand Risk	3.84	0.92
Risk Monitoring	4.09	0.63
Risk Mitigation	4.05	0.68
Supply Chain Performance	4.21	0.65

### Data normality via skewness and kurtosis

The highest values were -0.19 for skewness and 0.41 for kurtosis, respectively. According to Bajpai (2011), when employing SEM, acceptable skewness values range from -3 to +3, whereas acceptable kurtosis values range from 10 to +10. The findings demonstrate that the distribution data in this instance are essentially homogeneous Table 8.

**Table 8.** Normality Via Skewness and Kurtosis.

Constructs	Skewness	Std. Error	Kurtosis	Std. Error
Supply Risk	-0.788	0.172	0.410	0.342
Process Risk	-0.311	0.172	-0.489	0.342
Demand Risk	-0.735	0.172	0.173	0.342
Risk Monitoring	-0.421	0.172	-0.037	0.342
Risk Mitigation	-0.195	0.172	-0.552	0.342
Supply Chain Performance	-0.646	0.172	0.287	0.342

Source: Bajpai (2011).

### Measurement model analysis

In assessing the internal consistency and reliability of a measure, it’s essential for Factor Loading, Cronbach’s alpha and composite reliability (CR) to equal or exceed 0.70, and average variance extracted (AVE) to equal or surpass 0.50 ( Hair et al., 2017). The measurement model’s findings in this study reveal that all variables meet these criteria, with Factor Loading, Cronbach’s alpha and CR values surpassing 0.7, and AVE values exceeding 0.5 (see Table 9). These results indicate satisfactory reliability and internal consistency for the variables under consideration.

**Table 9.** Measurement item model assessment.

Construct	Item No	Factor Loading	Cronbach Alpha	CR	AVE
Demand Risk	DR1	0.785	0.916	0.937	0.750
	DR2	0.857			
	DR3	0.899			
	DR4	0.898			
	DR5	0.866			
Process Risk	PR1	0.846	0.950	0.961	0.833
	PR2	0.951			
	PR3	0.925			
	PR4	0.907			
	PR5	0.932			
Risk mitigation	RM1	0.833	0.831	0.887	0.664
	RM2	0.833			
	RM3	0.829			
	RM4	0.763			
Risk monitoring	RMI2	0.845	0.863	0.908	0.712
	RMI3	0.876			
	RMI4	0.887			
	RMI5	0.761			
Supply Risk	SR1	0.804	0.839	0.885	0.607
	SR2	0.800			
	SR3	0.738			
	SR4	0.788			
	SR5	0.767			
Supply Chain Performance	SCP1	0.825	0.876	0.910	0.669
	SCP2	0.859			
	SCP3	0.791			
	SCP4	0.810			
	SCP5	0.802			

**Note:** Items RM1 & RMI5 were deleted due to low loadings. CA=Cronbach’s α; CR=Composite Reliability, and AVE= Average of variance extracted; Supply Risk (SR), Process Risk (PR), Demand Risk (DR), Risk Monitoring (RM), Risk Mitigation (RMI), and Supply Chain Performance (SCP).

### Discriminant validity (Fornell–Larcker criterion)

The Fornell-Lacker criterion method for assessing discriminant validity compares the square root of the Average Variance Extracted (AVE) for each latent variable with the correlations between latent variables ( Hair et al., 2014). This method ensures that each latent variable primarily explains the variance within its own indicators rather than the variance of other latent variables. Consequently, in Table 10, the bolded diagonal elements (representing the square root of AVE) are consistently larger than the corresponding off-diagonal elements within their respective rows and columns, affirming discriminant validity.

**Table 10.** Fornell–Larcker criterion.

	DR	PR	RM	RMI	SCP	SR
DR	**0.866**					
PR	0.308	**0.913**				
RM	0.425	0.525	**0.815**			
RMI	0.371	0.372	0.753	**0.844**		
SCP	0.442	0.484	0.583	0.654	**0.818**	
SR	0.360	0.581	0.481	0.512	0.682	**0.779**

### Heterotrait-Monotrait (HTMT)

In addressing concerns related to discriminant validity within variance-based structural equation modeling, the heterotrait-monotrait ratio (HTMT) was introduced by Henseler et al. (2016) and supported by Voorhees et al. (2016). HTMT assesses the ratio of correlations within a trait to those between traits. According to Henseler et al. (2016), a benchmark value below 0.90 is recommended for evaluating discriminant validity using the HTMT criterion. As emphasized by Hair et al. (2017), discriminant validity is confirmed when HTMT values fall below 0.90. Elevated HTMT values suggest potential issues with discriminant validity, underscoring the importance of maintaining values below 0.9, as proposed by Henseler et al. (2015) and Henseler et al. (2016). Table 11 displays the HTMT values for all latent variables, indicating values lower than 0.9.

**Table 11.** Heterotrait-monotrait (HTMT).

	DR	PR	RM	RMI	SCP	SR
DR						
PR	0.323					
RM	0.485	0.582				
RMI	0.418	0.407	0.893			
SCP	0.492	0.525	0.678	0.752		
SR	0.413	0.633	0.561	0.593	0.788	

Note: Supply Risk (SR), Process Risk (PR), Demand Risk (DR), Risk Monitoring (RM), Risk Mitigation (RMI), and Supply Chain Performance (SCP).

### Structural Model

**Figure 2.**
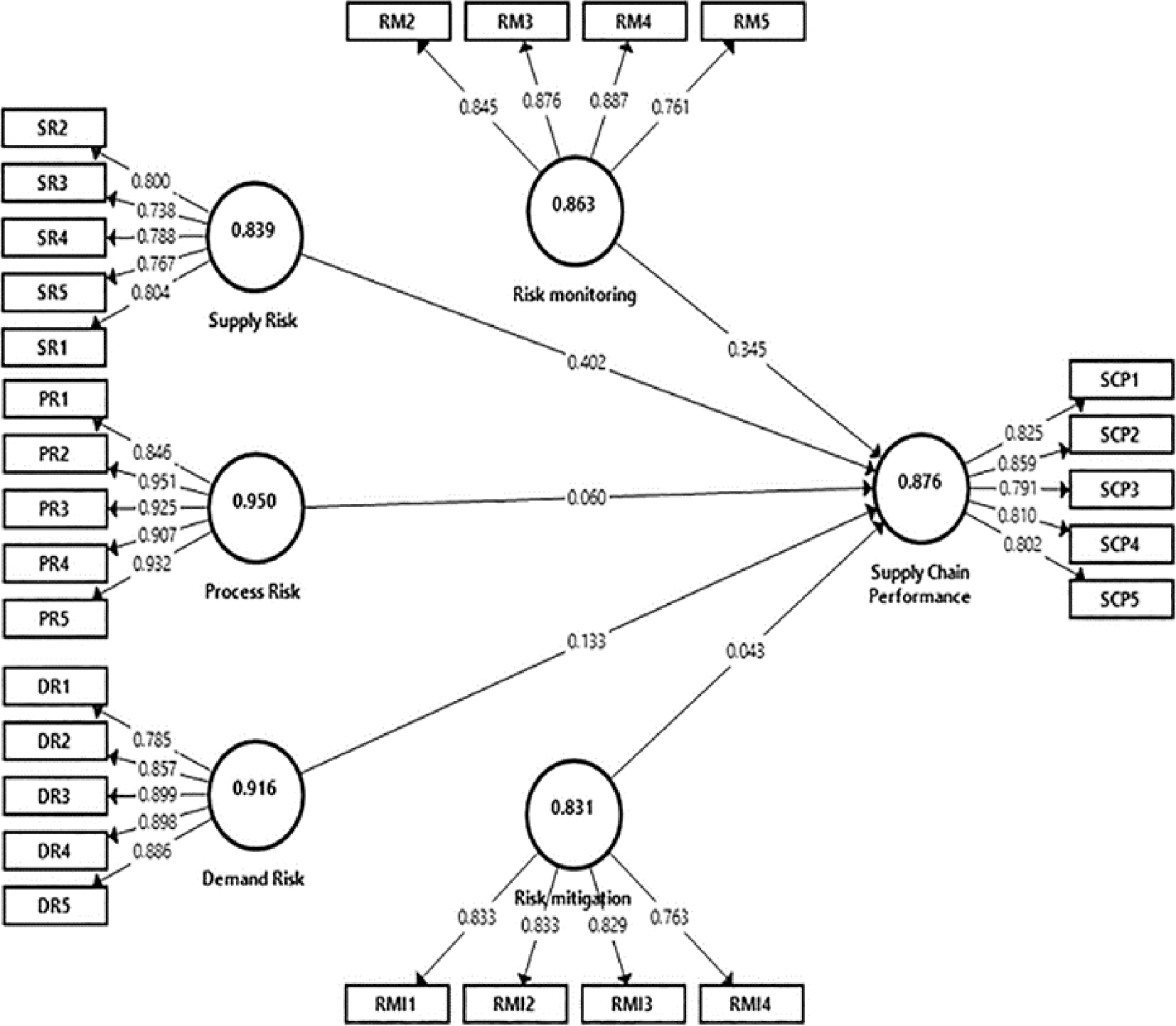
Measurement model illustration using PLS Algorithm. Source: Authors' original data.

Structural Model with the successful verification of the measurement model, attention can now shift towards confirming the structural model. This verification process is crucial for assessing the relationships between the constructs in the study model. model. The structural model elucidates these relationships, showcasing the interconnectedness of its components. Various measures, as outlined by Henseler, Hubona, and Ray (2016), were employed to evaluate the degree of interconnectedness within the structural indications within the structures were established reflectively. A diagram depicting Structural Equation Modeling showcases the assessment of supply chain performance through independent variables such as supply, process, demand risk capital, wherein risk monitoring and risk mitigation acts as a mediator. As illustrated in Figure 2, to enhance the reliability and validity of the constructs, items with low standardized loading factors (<0.7) were excluded.

### Lateral Collinearity Assessment Test

The assessment of lateral multicollinearity requires examining the inner Variance Inflation Factor (VIF) values of the independent variables. These values should ideally be below 5 and 3.3, as recommended by Hair et al. (2016) and Tabachnick and Fidell (2023). If these thresholds are met, it suggests no significant concerns regarding multicollinearity. Furthermore, to ascertain the absence of common method bias, all VIFs resulting from a comprehensive collinearity test should be below 5, as indicated in Table 12.

**Table 12.** Lateral Collinearity Assessment Test (VIF).

	Risk mitigation	Risk monitoring	Supply Chain Performance
Demand Risk	1.169	1.169	1.273
Process Risk	1.536	1.536	1.770
Risk mitigation			2.845
Risk monitoring			2.559
Supply Chain Performance	1.597	1.597	1.824

### Assessing Goodness-of-Fit Indices

Standardized Root Mean Square Residual (SRMR) lower values indicate better fit and common thresholds for good fit range from 0.05 to 0.08. Degree of Unweighted Least Squares (d_ULS) lower values suggest better model fit. There isn’t a widely accepted threshold for d_ULS, but it should be close to zero. Degree of Geodesic Discrepancy (d_G) lower values indicate better fit ( Kenny et al., 2015). A value close to zero is desirable. NFI values range from 0 to 1, where values closer to 1 indicate better fit. Commonly, a threshold of 0.90 or higher is considered indicative of good fit. By summarizing each index and comparing the obtained values to established thresholds, it can be confidently concluded that the model exhibits a good fit to the data Table 13.

**Table 13.** Model fit (Full Summary).

	Saturated model	Estimated model
SRMR	0.028	0.033
d_ULS	0.094	0.070
d_G	0.006	0.093
Chi-Square	219.654	252.220
NFI	0.721	0.683

Source: Kenny et al. (2015).

### R square (r ^2^)

Table 14 provides evidence that the R2 values for the variables Risk Mitigation, Risk Monitoring, and Supply Chain Performance are, respectively, 0.831, 0.863, and 0.876.

**Table 14.** R ^2^ summary.

	R ^2^	Adjusted R ^2^
Risk mitigation	0.831	0.829
Risk monitoring	0.863	0.861
Supply Chain Performance	0.876	0.876

### Effect size (f ^2^)

To evaluate the magnitude of impact for each effect within the route model, researchers can employ F-square ( Table 15) following Henseler’s (2018) recommendations. The effect size, denoted as f ^2^, is calculated by comparing the increase in R-squared to the proportion of unexplained variation in the endogenous latent variable. Henseler (2018) proposes that values of 0.35, 0.15, and 0.02 signify significant, moderate, and negligible effect sizes, respectively, according to the suggested criteria for magnitude assessment.

**Table 15.** Effect size f ^2^.

	Risk mitigation	Risk monitoring	Supply Chain Performance
Demand Risk	0.387	0.352	0.436
Process Risk	0.005	0.006	0. 115
Risk mitigation			0.002
Risk monitoring			0.118
Supply Chain Performance	0.039	0.138	0.232

### Assessment of predictive relevance (Q ^2^)

The cross-validated redundancy metric (Q ^2^) serves as a crucial indicator of predictive relevance within the framework of assessment recommended by Hair et al. (2011). This metric reflects the model’s predictive capability, with values anticipated to surpass zero, signaling the model’s aptness for prediction (see Table 16 for reference). Q-square values exceeding 0.20 or 0.25 are regarded as indicative of moderate to good predictive relevance. Researchers often rely on these thresholds to evaluate the model’s effectiveness in forecasting outcomes and informing decision-making processes.

**Table 16.** Construct Cross-Validated Redundancy.

	SSO	SSE	Q ^2^ (=1-SSE/SSO)
Risk mitigation	1172.00	893.76	0.237
Risk monitoring	1172.00	926.75	0.209
Supply Chain Performance	1465.00	884.90	0.396

Note: Q ^2^ < 0

### The path coefficient

**Figure 3.**
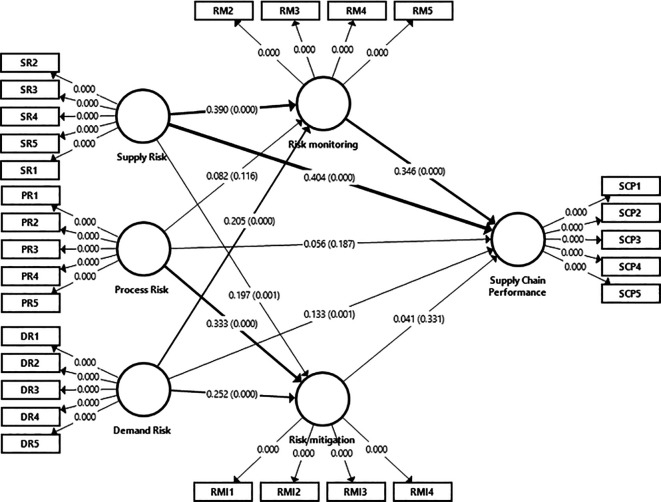
Structural model (Bootstrapping). Source: Authors' original data.

In evaluating the path coefficients linking constructs, the aim was to assess their support for the proposed hypotheses and the structural model. According to Hair et al. (2016), a minimum route coefficient value of 0.100 is deemed necessary for an impact to be considered within the model. Path coefficients were scrutinized to determine the relevance of the tested hypotheses between the constructs. Utilizing Smart-PLS bootstrapping and T-statistical analysis, the significance level of each route was established as shown in Figure 3. In the study, which involved a sample size of 385 responders and straightforward assumptions, significant values were observed at the 0.05 level. Examination of the path coefficient in Figure 3 and Table 17 unveiled relationships corresponding to hypotheses (H1a, H1b, H1c, H2c, H3a, H3b, H3c, H4) with significant values at the 0.05 level, whereas hypotheses H2a, H2b, and H5 were deemed non-significant.

**Table 17.** Path coefficient assessment.

Hypo	Relationships	*Beta*	*SD*	*t value*	*p value*	*Findings*
H1a	Supply Risk -> Supply Chain Perf	0.404	0.067	5.835	0.000	
H1b	Supply Risk -> Risk Monitoring	0.390	0.089	3.584	0.000	
H1c	Supply Risk -> Risk Mitigation	0.197	0.085	4.923	0.001	
H2a	Process Risk -> Supply Chain Perf	0.056	0.391	1.278	0.187	Not Supported
H2b	Process Risk -> Risk Monitoring	0.082	0.269	1.396	0.116	Not Supported
H2c	Process Risk -> Risk mitigation	0.333	0.057	3.981	0.000	*Supported* **
H3a	Demand Risk -> Supply Chain Perf	0.133	0.063	3.769	0.001	*Supported* **
H3b	Demand Risk -> Risk Monitoring	0.205	0.061	5.469	0.000	*Supported* **
H3c	Demand Risk -> Risk mitigation	0.252	0.051	4.923	0.000	*Supported* **
H4	Risk Monitoring -> Supply Chain Perf	0.346	0.085	4.217	0.000	Supported **
H5	Risk mitigation -> Supply Chain Perf	0.041	0.495	1.087	0.331	Not Supported

** Supported= P value <0.05.

Bootstrapping in the context of the PLS structural model is a resampling technique used to estimate the precision of the model’s parameter estimates. It involves repeatedly sampling (with replacement) from the data set and estimating the model for each sample. This process generates a distribution of estimates, which can be used to assess the stability and reliability of the path coefficients, loadings, and other statistics in the model. Bootstrapping provides confidence intervals and significance testing for the structural paths, helping researchers determine whether the relationships between constructs are statistically significant as shown in Figure 3 ( Hair et al., 2017; Henseler et al., 2009).

## Discussion

### Supply chain risks associated with consumer products

Businesses in the consumer goods sector recognize supplier, process, and demand risks as pivotal challenges shaping their supply chains ( Qiao & Zhao, 2023). Process risks arise from logistics inadequacies, inaccurate forecasting, and reliance on labor agreements. Supply and demand risks can disrupt operations due to discrepancies between supply and market demand. Weather’s impact on the economy, coupled with government regulations, exposes workplaces to environmental concerns, affecting production, sales, and transportation. Consumer goods entrepreneurs face heightened demand and financial risks compared to contract counterparts, attributed to limited resources ( Prabhu and Srivastava, 2023). Financial disruptions profoundly affect business operations, emphasizing the need for tailored financial solutions, especially for SMEs. The study reiterates the severity of supply chain risks, shedding light on their diverse impacts, underscoring the imperative for effective risk management strategies in the consumer goods sector.

### Supply chain risks and supply chain performance in consumer products

The second research objective of this study focuses on exploring the correlation between supply chain risks and supply chain performance within the consumer products sector. The findings underscore the significant impact of supply chain efficiency and risk on the ASEAN consumer products industry. The study addresses an empirical gap by establishing a clear link between supply chain risks and performance. While similar studies have made comparable assertions regarding this relationship, the research stands out for its robust analysis, particularly within the often-overlooked sector of consumer products ( Aghabegloo et al., 2024). Previous research predominantly concentrated on the manufacturing industry and primarily assessed process risks using metrics such as variance, lead time order fulfillment, and production specification ( Bokrantz & Dul, 2023; Rashid et al., 2024). However, process risk has received comparatively less attention than other forms of risk and warrants expansion from supplier-centric assessments to encompass the entire supply chain, extending from enterprises to retailers. In this study, the evaluation of supply risk was broadened to encompass foreign suppliers, yielding valuable insights into its impact on the quality, cost-efficiency, flexibility, and responsiveness of consumer products entrepreneurs. Thus, this research provides a comprehensive understanding of how supply chain risks influence overall performance within the consumer products industry.

### Risk monitoring mediates the relationship between management of supply chain risks and performance

The research findings highlight the crucial mediating role of risk monitoring in the relationship between supply chain performance and risk management. Rooted in the transaction cost concept, it’s evident that risk emerges from uncertain conditions. This study underscores that existing supply chain risks exert a substantial influence on the overall functionality of the supply chain, particularly within the highly unpredictable landscape of consumer products. Businesses operating in the consumer goods sector, especially those dealing with fresh produce, are particularly vulnerable to supply chain risks due to factors like high perishability, short shelf life, and complex engagement with various suppliers and purchasers ( Prashar & Vijaya, 2024). Effective risk management becomes imperative for enhancing supply chain performance in the face of significant supply chain risks. One of the primary objectives of this research was to examine the relationship between supply chain risks and the practices of supply chain risk management. In doing so, the study integrated perspectives from both the supply chain governance structure and the transaction cost theory. According to the transaction cost theory, there exists a strong connection between governance systems and the levels of uncertainty within a supply chain. This research delved into assessing supply chain risks within the framework of three key risk management pillars: risk identification, risk assessment, and risk mitigation approaches. Despite the acknowledged challenges in risk detection skills among entrepreneurs in the consumer products sector, the findings of this study reaffirm the significant impact of supply chain risks on business operations. To further enhance the theoretical foundation, integrating the insights from Rashid et al. (2024) can provide a more comprehensive understanding of the role of information processing and digital supply chains in bolstering supply chain resilience through effective risk management. Their study emphasizes the significance of information processing capabilities and digital supply chain integration in managing risks and improving resilience, which aligns with the current study’s focus on risk monitoring and mitigation. By incorporating these perspectives, the research can offer a more robust framework for understanding and addressing supply chain risks in the consumer goods sector.

### Risk mitigation mediates the relationship between management of supply chain risks and performance

The research outcomes also suggest a notable mediating role of risk mitigation in bridging the link between performance and supply chain risk management. The findings highlight that among the three components comprising supply chain risk management, only risk assessment and risk mitigation act as mediators in the relationship between supply chain risk and performance. Conversely, it was observed that risk mitigation did not exert a mediating influence on supply chain performance. The investigation reveals that merely identifying risks within the supply chain doesn’t positively affect performance; instead, it’s the evaluation of risks and the implementation of mitigation measures that lead to improved performance ( Han & Um, 2024; Li et al., 2024). This sheds light on why the identification phase of risk management might not efficiently establish the link between supply chain risk and performance. Previous research on supply chain risk has often overlooked or lacked theoretical justification for incorporating risk management strategies as mediating factors ( Altay & Pal, 2023). However, the current study supports the hypothesis that effective supply chain risk management serves as an intermediary to enhance business efficiency. This notion is theoretically and empirically validated by the study, demonstrating that elements of supply chain risk management, particularly risk assessment and mitigation strategies, significantly mediate the relationship between supply chain risks and performance, specifically within the realm of consumer products entrepreneurship ( Shishodia et al., 2023).

### Theoretical implication

Supply Risk includes risks related to supplier reliability, quality issues, disruptions, etc. Theoretical contributions from SCM theory suggest that managing supply risks effectively can lead to improved operational performance, cost efficiency, and resilience in the supply chain. Process risk includes process inefficiencies, breakdowns, bottlenecks, etc. Theoretical contributions may suggest that effective process management leads to improved agility, flexibility, and responsiveness within the supply chain, contributing to overall performance ( Bokrantz & Dul, 2023). Demand risk pertains to uncertainties and variability in customer demand. Fluctuations in demand patterns, unexpected changes in customer preferences, or market dynamics can all contribute to demand risk. Theoretical contributions highlight the importance of demand forecasting, customer relationship management, and market intelligence in mitigating demand risks and improving supply chain performance ( Nurdiana et al., 2023). Risk monitoring involves the continuous surveillance and assessment of various risks within the supply chain. It includes activities such as data collection, analysis, early warning systems, etc ( Jeong & Chung, 2023). Theoretical contributions suggest that proactive risk monitoring enables timely identification of potential threats, facilitating prompt decision-making and risk mitigation actions. Risk mitigation refers to the strategies and actions taken to reduce the impact or likelihood of risks materializing within the supply chain. This can include diversification of suppliers, inventory buffers, contingency planning, etc. SCM theory emphasizes the importance of risk mitigation as a proactive approach to safeguarding supply chain performance and resilience. Theoretical contributions in SCM may emphasize that effective risk management practices positively influence supply chain performance by enhancing operational efficiency, reducing disruptions, improving customer service levels, and ultimately, contributing to competitive advantage and organizational success ( Rimin et al., 2021). SCM theory underscores the significance of addressing supply, process, and demand risks within the supply chain context and highlights the mediating role of risk monitoring and risk mitigation in enhancing supply chain performance ( Solijonov et al., 2023). By empirically testing this model, researchers can further validate the theoretical underpinnings and provide practical insights for managers to better manage risks and improve supply chain performance in dynamic and uncertain environments. Additionally, this model emphasizes the importance of a holistic and integrated approach to risk management, aligning with the overarching goals of SCM theory.

### Practical implication

By categorizing risks into supply, process, and demand categories, supply chain managers can better understand and prioritize potential threats to their operations. This allows for a more targeted approach to risk management, ensuring that resources are allocated efficiently to mitigate the most critical risks ( Halkier & James, 2022). Recognizing risk monitoring and mitigation as mediating variables underscores their importance in proactive risk management. Practically, this implies implementing robust systems for monitoring supply chain activities, gathering relevant data, and utilizing advanced analytics tools for risk detection and assessment. It also involves developing and implementing effective risk mitigation strategies tailored to the specific types of risks identified, such as diversifying suppliers, establishing contingency plans, or investing in technology to improve process resilience ( Li et al., 2024). The ultimate goal of supply chain management is to enhance performance, and the model acknowledges this by considering supply chain performance as the dependent variable. Practically, supply chain managers can use performance metrics such as on-time delivery, inventory turnover, and customer satisfaction to assess the effectiveness of their risk management efforts. By continually monitoring and evaluating performance in relation to risk management activities, managers can identify areas for improvement and implement targeted initiatives to optimize supply chain performance. The model suggests a strong coordination among supply chain partners and stakeholders, emphasizing the importance of collaboration in risk assessment and management. Practically, this involves fostering open communication channels, sharing information and insights, and establishing collaborative relationships with suppliers, customers, and other key stakeholders ( Meltzer, 2024). By working together to identify and address risks, supply chain partners can enhance their collective resilience and responsiveness to potential disruptions. Effective risk management practices contribute to building resilience and sustainability within the supply chain. By proactively identifying and mitigating risks, supply chain managers can minimize the impact of disruptions, improve operational stability, and ensure continuity of supply. This not only enhances short-term performance but also strengthens the long-term viability and competitiveness of the supply chain. Overall, the model provides a practical framework for supply chain managers to assess, monitor, and mitigate risks effectively, ultimately leading to improved supply chain performance and resilience in dynamic and uncertain environments. By aligning with supply chain management theory, it offers actionable insights and guidelines for practitioners to enhance their risk management practices and drive competitive advantage in today’s complex business landscape. Business owners in the consumer products industry may use these specifics as a reference to better understand their supplier networks. Entrepreneurs in the consumer products industry need to acquire, transform, and use knowledge in order to compete in the market. Owners of consumer products companies who have open lines of communication with their supply chain partners will be better able to identify obstacles and quickly adapt their strategy to deal with supply chain risks ( Pangestuti et al., 2023). Lowering risk will influence tactics and boost customer happiness, both of which will enhance the productivity of business owners of consumer products firms.

## Conclusion

This study underscores the critical role of effective supply chain risk management in enhancing the performance of businesses in the consumer products sector, particularly in the face of uncertainties inherent in supply, process, and demand risks. It establishes a clear link between supply chain risks and performance, highlighting that proactive risk monitoring and mitigation strategies are essential to business resilience. The findings reveal that risk monitoring and mitigation play crucial mediating roles in this relationship, enabling businesses to adapt to disruptions and improve overall supply chain efficiency. A significant contribution of this research is the emphasis on continuous improvement in risk management practices. The study demonstrates that risk management should not be viewed as a one-time activity but as an ongoing process that requires regular evaluation and adaptation. Entrepreneurs, especially in the fresh produce sector, must continuously refine their risk assessment and mitigation strategies to stay ahead of emerging risks and ensure long-term business success. Furthermore, this research provides practical insights for supply chain managers to develop more robust risk management systems, emphasizing the need for real-time monitoring, collaboration with supply chain partners, and the implementation of flexible risk mitigation strategies. By fostering a culture of continuous improvement, businesses can better respond to dynamic market conditions, reduce vulnerabilities, and enhance their competitive advantage. The findings of this study reinforce the importance of a holistic and adaptive approach to supply chain risk management. By integrating ongoing risk monitoring and mitigation efforts, businesses in the consumer products industry can not only survive disruptions but also thrive in a volatile and uncertain business environment.

### Limitations

This study acknowledges several limitations that arise from the diverse characteristics of the ASEAN region. The varied industries and supply chain structures across ASEAN member states present challenges, with each country’s unique economic, regulatory, and infrastructural context influencing supply chain dynamics differently. The limited availability and reliability of data further hinder accurate risk assessment and the development of effective supply chain management strategies. Cultural and language barriers also complicate data collection and analysis, while the diversity of consumer products and market dynamics makes risk identification more complex. The prevalence of informal economies introduces additional challenges, such as counterfeit products and labor exploitation, which impact supply chain operations. Furthermore, the fragmented nature of supply chains and the dominance of small enterprises limit effective networking opportunities. Cross-border trade complexities and regulatory differences hinder collaboration and information sharing, particularly for transnational risks like natural disasters and political instability, which further complicate risk mitigation efforts.

### Suggestions for future research

Future research on supply chain management (SCM) in ASEAN countries should prioritize identifying and prioritizing critical risks across geopolitical, environmental, economic, and technological domains. Studies should analyze individual member states’ challenges and assess the impact of regional integration efforts like the ASEAN Economic Community (AEC) on supply chain vulnerabilities. Utilizing diverse methodologies, researchers can tailor risk assessment frameworks to ASEAN’s varied economies. Collaborative research initiatives involving academia, industry, and government can exchange best practices in risk mitigation. Exploring evolving consumer preferences, market trends, and regulatory frameworks is crucial, as is studying successful risk mitigation practices in the consumer goods industry. Research on government policies, industry standards, and international collaborations in promoting supply chain resilience is needed. Comparative analyses can identify best practices for building resilient supply chains in ASEAN. Longitudinal studies tracking supply chain risks in response to geopolitical tensions, trade disruptions, and technological advancements are also warranted. By considering internal and external risk factors, researchers can offer recommendations for enhancing ASEAN’s consumer products industry’s risk management capabilities.

## Ethical approval statement

Hereby, I, Kalisri Logeswaran Aravindan, consciously assure that for the manuscript Resilience in the Face of Uncertainty: Navigating Supply Chain Challenges Through Proactive Risk Surveillance and Mitigation Strategies among SMEs in ASEAN countries the following is fulfilled:

1) This material is the authors own original work, which has not been previously published elsewhere.
2) The paper is not currently being considered for publication elsewhere.
3) The paper reflects the author’s own research and analysis in a truthful and complete manner.
4) The paper properly credits the meaningful contributions of co-authors and co-researchers.
5) The results are appropriately placed in the context of prior and existing research.
6) All sources used are properly disclosed (correct citation). Literally copying of text is indicated by using quotation marks and giving proper reference.
7) All authors have been personally and actively involved in substantial work leading to the paper and will take public responsibility for its content.

The violation of the Ethical Statement rules may result in severe consequences.

This study has been endorsed by SEGi (RMIC) approval committee was obtained from the Institutional Review Board (IRB) of SEGi University with the approval number IRB-2023-14532, granted on May 5, 2023.

1. Sanmugam Annamalah
2. Kalisri Logeswaran Aravindan
3. Selim Ahmed

We agree with the above statements and declare that this submission follows the policies of as outlined in the Guide for Authors and in the Ethical Statement.

Date: 11/5//2023

Corresponding author’s signature: Kalisri Logeswaran Aravindan

## Ethical statement

We, the undersigned co-authors, affirm that our manuscript titled “Resilience in the Face of Uncertainty: Navigating Supply Chain Challenges Through Proactive Risk Surveillance and Mitigation Strategies among SMEs in ASEAN Countries” contains original material that has not been published, either in full or in part, in any other journal or publication. This manuscript is not under consideration for publication elsewhere. Each author has made significant and active contributions to the research and preparation of this manuscript and accepts joint and individual responsibility for its content.

This study was conducted in accordance with the ethical principles outlined in the Declaration of Helsinki. Ethical approval for the research was obtained from the Institutional Review Board (IRB) of SEGi University with the approval number IRB-2023-14532, granted on May 5, 2023.

Participants were provided with comprehensive information about the study, including its purpose, procedures, potential risks, and benefits. Written informed consent was obtained from all participants prior to their inclusion in the study. They were assured that their participation was voluntary and that they could withdraw at any time without any consequences.

The IRB reference number for this approval is IRB-2023-14532.

## Consent to participate

Informed verbal consent was obtained from all participants involved in this study. Verbal consent was deemed appropriate due to participants’ literacy levels, cultural considerations, etc. However participants received comprehensive information about the study’s purpose, procedures, potential risks, and benefits. They were assured of their right to withdraw at any time without any repercussions.

Date: 11/5/2023

**Corresponding author:** Kalisri Logeswaran Aravindan

## Participant consent form

Research Project Title: Resilience in the Face of Uncertainty: Navigating Supply Chain Challenges Through Proactive Risk Surveillance and Mitigation Strategies among SMEs in ASEAN countries.

To be completed by the participant.

**Table:** 

• I have read the information sheet about this study. • I have had an opportunity to ask questions and discuss this study. • I have received satisfactory answers to all my questions. • I have received enough information about this study. • I understand that I am/the participant is free to withdraw from this study: ○ At any time (until such date as this will no longer be possible, which I have been told) ○ Without giving a reason for withdrawing • I understand that my research data may be used for a further project in anonymous form, but I am able to opt out of this if I so wish, by ticking here. • I agree to take part in this study.	
Signed (participant)	Date
Name in block letters	
Signed	Date
Name in block letters	
Signature of researcher	Date
This project is supervised by:	
Researcher’s contact details (including telephone number and e-mail address):	

## Data Availability

**Figshare:** Resilience in the Face of Uncertainty: Navigating Supply Chain Challenges Through Proactive Risk Surveillance and Mitigation Strategies among SMEs in ASEAN countries, https://doi.org/10.6084/m9.figshare.25683687.v3 ( Annamalah, 2024). **Data license:** CC BY 4.0
