# Supplementary material for: Resilience in the Face of Uncertainty: Navigating Supply Chain Challenges Through Proactive Risk Surveillance and Mitigation Strategies among SMEs in ASEAN countries
Source: F1000Res. 2025 Jun 24;13:1037. Originally published 2024 Sep 10. [Version 2] doi: 10.12688/f1000research.153654.2 (PMC12411842; doi:10.12688/f1000research.153654.2)
Supplement: Peer review history

# Reviewer report: Reviewer response for version 2

**DOI:** 10.5256/f1000research.183583.r409516

**Published:** 2025-09-04

**Authors:** Muzaffar Asad

**Affiliations:** 1 Tecnologico de Monterrey, Monterrey, Nuevo Leon, Mexico

Dear Researcher,

 The work that you have chosen is very good and timely. I have seen that you have identified the important aspects for SMEs regarding supply chain risk management. The introduction section contains very new sources which is a very good thing, however, I have realized that you have overlooked some important research works which must be reviewed and added to the research. When you review the work, you will realize how you can add novelty in your research, therefore, I believe that you must consult to make the paper equipped with more current research in the field and criticize the existing literature to provide solid justification for the work you have conducted. 

 1. Asad, M. (2024). Impact of environmental management on sustainable performance of Pakistani entrepreneurial firms: The mediating role of green product innovation and the moderating effect of transformational leadership. *Sustainability, 16*(24), 10935. doi:https://doi.org/10.3390/su162410935

 2. Awain, A. M., Asad, M., Sulaiman, M. A., Asif, M. U., & Shanfari, K. S. (2025). Impact of supply chain risk management on product innovation performance of Omani SMEs: Synergetic moderation of technological turbulence and entrepreneurial networking. *Sustainability, 17*(7), 2903. doi:https://doi.org/10.3390/su17072903

 the above papers will guide you to answers the critical questions like why SMEs, why Supply Chain Risk Management. 

 In the methodology section please have a look at the papers to confirm the sampling criteria which your methodology is lacking. answer Why ASEAN region. Likewise, why the sector why the respondents all such answers should be given for which these papers will guide you.

 3. Sulaiman, M. A., Asad, M., Awain, A. M., Asif, M. U., & Shanfari, K. S. (2024). Entrepreneurial marketing and performance: Contingent role of market turbulence. *Discover Sustainability, 5*, 1-20. doi:https://doi.org/10.1007/s43621-024-00710-8. 

 Moreover, in the discussion and conclusions section you have identified the link between your study and other findings. However, there is a need to clearly identify the theoretical significance of the study and practical or managerial significance of the study. If you link your findings with the latest research and show how your study contributes to the literature, it gives the look that you have addressed the current issue and have conducted a Novel work, instead of addressing an old work. By Addressing the above-mentioned suggestions, I believe that you can easily make the Paper worth indexing this Journal.

Is the work clearly and accurately presented and does it cite the current literature?

Partly

If applicable, is the statistical analysis and its interpretation appropriate?

Yes

Are all the source data underlying the results available to ensure full reproducibility?

Partly

Is the study design appropriate and is the work technically sound?

Yes

Are the conclusions drawn adequately supported by the results?

Yes

Are sufficient details of methods and analysis provided to allow replication by others?

Partly

Reviewer Expertise:

Entrepreneurship, SMEs, Marketing, Supply chain, Big Data.

I confirm that I have read this submission and believe that I have an appropriate level of expertise to confirm that it is of an acceptable scientific standard, however I have significant reservations, as outlined above.

## References

[1] : Impact of Environmental Management on Sustainable Performance of Pakistani Entrepreneurial Firms: The Mediating Role of Green Product Innovation and the Moderating Effect of Transformational Leadership. *Sustainability* .2024;16(24) : 10.3390/su162410935 10.3390/su162410935

[2] : Impact of Supply Chain Risk Management on Product Innovation Performance of Omani SMEs: Synergetic Moderation of Technological Turbulence and Entrepreneurial Networking. *Sustainability* .2025;17(7) : 10.3390/su17072903 10.3390/su17072903

[3] : Entrepreneurial marketing and performance: contingent role of market turbulence. *Discover Sustainability* .2024;5(1) : 10.1007/s43621-024-00710-8 10.1007/s43621-024-00710-8

# Reviewer report: Reviewer response for version 2

**DOI:** 10.5256/f1000research.183583.r394392

**Published:** 2025-09-04

**Authors:** Aamir Rashid

**Affiliations:** 1 York College,, City University of New York (CUNY), New York, New York, USA

Thank you for the revisions. I have read the paper carefully and paid particular attention to the theoretical aspects. However, I could not clearly identify to which theory or theories this research is contributing. Although the authors include a section titled *“Underpinning Theory”*, the content in this section appears more like an extended literature review and explanation of concepts such as risk mitigation, rather than an explicit reference to a specific theory and its integration into this research.

 Similarly, in the *“Theoretical Implications”* section, the authors refer broadly to “SCM Theory.” It is not clear whether “SCM Theory” constitutes a recognized and established theory. This requires clarification.

 I recommend revisions to both sections to explicitly identify the guiding theory or theories, integrate them into the study framework, and clearly articulate the contribution of this research to those theories. Moreover, the literature should be more explicitly linked with the chosen theory/theories, which is currently missing in this revised manuscript.

 Further, the insignificant hypotheses need further explanation. These relationships can potentially provide meaningful contributions to the theoretical lens applied in the study and therefore should not be overlooked.

 Lastly, the 0.538 Cronbach's Alpha value for Risk Monitoring is below 0.70 (recommended value). The authors can explain the reason and justification to satisfy these values. Or they can provide reference(s) that show the 0.538 value is sufficient, etc.

Is the work clearly and accurately presented and does it cite the current literature?

Yes

If applicable, is the statistical analysis and its interpretation appropriate?

Yes

Are all the source data underlying the results available to ensure full reproducibility?

Yes

Is the study design appropriate and is the work technically sound?

Partly

Are the conclusions drawn adequately supported by the results?

Yes

Are sufficient details of methods and analysis provided to allow replication by others?

Yes

Reviewer Expertise:

Supply Chain Managment, Logistics Management, Operations Management

I confirm that I have read this submission and believe that I have an appropriate level of expertise to confirm that it is of an acceptable scientific standard, however I have significant reservations, as outlined above.

# Reviewer report: Reviewer response for version 2

**DOI:** 10.5256/f1000research.183583.r394393

**Published:** 2025-08-09

**Authors:** Wilson Kosasih

**Affiliations:** 1 Tarumanagara University, West Jakarta, Jakarta, Indonesia

Dear Editor,

 I confirm that the manuscript has been thoroughly revised according to my comments and reviews. The authors have addressed all the concerns and suggestions, and the manuscript has been significantly improved.

 Given the quality and relevance of the research, I highly recommend indexing this article as one of the references in the field of resilience and risk supply chain management in ASEAN countries.

 Congratulations !!! 

 Best regards,

 Dr. Ir. Wilson Kosasih

Is the work clearly and accurately presented and does it cite the current literature?

Partly

If applicable, is the statistical analysis and its interpretation appropriate?

Yes

Are all the source data underlying the results available to ensure full reproducibility?

Partly

Is the study design appropriate and is the work technically sound?

Yes

Are the conclusions drawn adequately supported by the results?

Partly

Are sufficient details of methods and analysis provided to allow replication by others?

Yes

Reviewer Expertise:

the supply chain and sustainability, lean and green practices

I confirm that I have read this submission and believe that I have an appropriate level of expertise to confirm that it is of an acceptable scientific standard.

# Reviewer report: Reviewer response for version 1

**DOI:** 10.5256/f1000research.168575.r381941

**Published:** 2025-05-02

**Authors:** Aamir Rashid

**Affiliations:** 1 York College,, City University of New York (CUNY), New York, New York, USA

Thank you for this wonderful research. My brief comments are as follows:

1. The study references risk management literature. It alludes to frameworks like transaction cost theory but does not rigorously apply or develop any clear theoretical framework to guide hypothesis development or analysis. The mention of supporting studies appears more as background context than theoretical scaffolding. The following study may help the authors’ theoretical background: Rashid A, et al., 2024 (Ref 1)
2. Insufficient explanation of construct development, item selection, or justification of the mediators. Risk constructs are treated as self-evident rather than grounded in validated models. Furthermore, low reliability (Cronbach’s alpha for risk monitoring at 0.538) suggests measurement concerns.
3. Literature is often paraphrased without synthesis, which dilutes analytical clarity and reader engagement.
4. The study collects data from 385 SMEs across 10 ASEAN countries but lacks meaningful cross-country comparison or analysis. It treats the ASEAN region as homogenous, which weakens contextual specificity.

Is the work clearly and accurately presented and does it cite the current literature?

Yes

If applicable, is the statistical analysis and its interpretation appropriate?

Yes

Are all the source data underlying the results available to ensure full reproducibility?

Yes

Is the study design appropriate and is the work technically sound?

Partly

Are the conclusions drawn adequately supported by the results?

Yes

Are sufficient details of methods and analysis provided to allow replication by others?

Yes

Reviewer Expertise:

Supply Chain Managment, Logistics Management, Operations Management

I confirm that I have read this submission and believe that I have an appropriate level of expertise to confirm that it is of an acceptable scientific standard, however I have significant reservations, as outlined above.

## References

[1] : Role of information processing and digital supply chain in supply chain resilience through supply chain risk management. *Journal of Global Operations and Strategic Sourcing* .2024;17(2) : 10.1108/JGOSS-12-2023-0106 429-447 10.1108/JGOSS-12-2023-0106

# Reviewer report: Reviewer response for version 1

**DOI:** 10.5256/f1000research.168575.r339835

**Published:** 2024-12-02

**Authors:** Wilson Kosasih

**Affiliations:** 1 Tarumanagara University, West Jakarta, Jakarta, Indonesia

In this paper, the authors presented a survey-based study that aims to explore the relationship between supply chain risks and supply chain performance in the consumer products sector within ASEAN nations. Specifically, it investigates whether risk monitoring and risk mitigation serve as mediators in this relationship. In my opinion, this paper can be considered for approval, after taking into account the following remarks:

1. In Table 1, please check the no. of Indonesian SMEs again. The context is different from MSMEs. The number should be around 864,000 SMEs.
2. Please present the latent variables and their indicators in detail.
3. In the abstract, the authors mentioned that the study utilized a sample of 385 entrepreneurs from the consumer products industry in ASEAN countries. But in sampling design section, they mentioned the simple random sampling technique was utilized to select participants. What does it means ?
4. In the conclusion, the authors wrote like follow: …. “ *Overall, the findings emphasize the importance of continuous improvement in supply chain risk management for entrepreneurs in the consumer products industry*.”…. This statement needs to be consolidated and presented or discussed in depth in the discussion section.
5. The English language should be verified.

Is the work clearly and accurately presented and does it cite the current literature?

Partly

If applicable, is the statistical analysis and its interpretation appropriate?

Yes

Are all the source data underlying the results available to ensure full reproducibility?

Partly

Is the study design appropriate and is the work technically sound?

Yes

Are the conclusions drawn adequately supported by the results?

Partly

Are sufficient details of methods and analysis provided to allow replication by others?

Yes

Reviewer Expertise:

the supply chain and sustainability, lean and green practices

I confirm that I have read this submission and believe that I have an appropriate level of expertise to confirm that it is of an acceptable scientific standard, however I have significant reservations, as outlined above.
